# Contrasting population genetic responses to migration barriers in two native and an invasive freshwater fish

**DOI:** 10.1111/eva.13469

**Published:** 2022-11-05

**Authors:** Io S. Deflem, Federico C. F. Calboli, Henrik Christiansen, Bart Hellemans, Joost A. M. Raeymaekers, Filip A. M. Volckaert

**Affiliations:** ^1^ Laboratory of Biodiversity and Evolutionary Genomics KU Leuven Leuven Belgium; ^2^ Natural Resources Institute Finland (Luke) Jokioinen Finland; ^3^ Faculty of Biosciences and Aquaculture Nord University Bodø Norway

**Keywords:** conservation, invasive species, migration barriers, population genomics, riverine fish, riverscape

## Abstract

Habitat fragmentation impacts the distribution of genetic diversity and population genetic structure. Therefore, protecting the evolutionary potential of species, especially in the context of the current rate of human‐induced environmental change, is an important goal. In riverine ecosystems, migration barriers affect the genetic structure of native species, while also influencing the spread of invasive species. In this study, we compare genetic patterns of two native and one highly invasive riverine fish species in a Belgian river basin, namely the native three‐spined stickleback (*Gasterosteus aculeatus*) and stone loach (*Barbatula barbatula*), and the non‐native and invasive topmouth gudgeon (*Pseudorasbora parva*). We aimed to characterize both natural and anthropogenic determinants of genetic diversity and population genetic connectivity. Genetic diversity was highest in topmouth gudgeon, followed by stone loach and three‐spined stickleback. The correlation between downstream distance and genetic diversity, a pattern often observed in riverine systems, was only marginally significant in stone loach and three‐spined stickleback, while genetic diversity strongly declined with increasing number of barriers in topmouth gudgeon. An Isolation‐By‐Distance pattern characterizes the population genetic structure of each species. Population differentiation was only associated with migration barriers in the invasive topmouth gudgeon, while genetic composition of all species seemed at least partially determined by the presence of migration barriers. Among the six barrier types considered (watermills, sluices, tunnels, weirs, riverbed obstructions, and others), the presence of watermills was the strongest driver of genetic structure and composition. Our results indicate that conservation and restoration actions, focusing on conserving genetic patterns, cannot be generalized across species. Moreover, measures might target either on restoring connectivity, while risking a rapid spread of the invasive topmouth gudgeon, or not restoring connectivity, while risking native species extinction in upstream populations.

## 1 INTRODUCTION

Anthropogenic alterations of the environment significantly impact freshwater ecosystems worldwide, causing a drastic decline in both species and genetic diversity (Dudgeon et al., 2006; Leigh et al., 2019). Habitat fragmentation, resulting from the construction of artificial barriers such as watermills and dams, has been identified as an important cause of the decline of riverine biodiversity (Dudgeon et al., 2006; Fullerton et al., 2010; Nilsson et al., 2005). The construction of barriers reduces available habitat and isolates populations, leading to a steep decline in both neutral and adaptive genetic diversity (Henle et al., 2004; Horreo et al., 2011). Yet, spatial patterns of genetic diversity remain poorly documented and are not often incorporated in conservation planning, although the importance for the persistence and resilience of species has been recognized for long (Hoban et al., 2020; Laikre et al., 2020; Taberlet et al., 2012). Hence, understanding natural and anthropogenic spatial drivers of genetic structure and connectivity is essential to prevent further biodiversity loss and to identify relevant restoration measures (Manel & Holderegger, 2013; Paz‐Vinas & Blanchet, 2015).

It is well understood that the unique physical structure of riverine systems shapes micro‐evolutionary processes such as migration, drift, and selection (Altermatt, 2013; Fourcade et al., 2013; Manel et al., 2020; Thomaz et al., 2016). The movement and dispersal of riverine fauna is strongly constrained by the dendritic network of rivers, a process that is reinforced by the unidirectional flow of water (Altermatt, 2013; Peterson et al., 2013). This restricted dispersal reduces gene flow, which in turn generates unique spatial patterns of genetic diversity and divergence (Altermatt, 2013; Ronce, 2007). Patterns commonly reported in riverine systems include a downstream increase in genetic diversity (DIGD; Alp et al., 2012; Kikuchi et al., 2011; Torterotot et al., 2014), correlations between genetic diversity, effective population size, and upstream basin size (Hänfling et al., 2004), and patterns resulting from Isolation‐By‐Distance (IBD; Primmer et al., 2006). In addition, empirical and theoretical studies indicate that network connectivity is a key component shaping patterns of genetic variation (Labonne et al., 2008; Paz‐Vinas & Blanchet, 2015; Thomaz et al., 2016).

Human activities influence natural riverine spatial patterns by the construction of migration barriers (e.g. watermills, dams, and weirs), which significantly decreases population connectivity. Barriers isolate populations from one another by reducing the number of migrants and effective population sizes. Small (effective) populations are more vulnerable to stochastic events and inbreeding, which in turn results in the loss of genetic diversity (Fischer & Lindenmayer, 2007; Frankham, 2010). Reduced genetic diversity compromises the ability of populations to respond and adapt to environmental change, increasing population vulnerability and eventually affecting the extinction risk of an entire species (Frankham, 2005; Spielman et al., 2004; Stockwell et al., 2003).

Although man‐made barriers are general drivers of genetic structure in riverine fishes (e.g. Brauer & Beheregaray, 2020; Faulks et al., 2011; Raeymaekers et al., 2008; Roberts et al., 2013; Wofford et al., 2005), few studies have compared the effects between species (e.g. Blanchet et al., 2010; Prunier et al., 2018), and none have focused on the effect on invasive species. Responses to habitat fragmentation are highly species specific, and variation has been attributed to dispersal capacity, body size, historical population size and structure, and trophic status (Blanchet et al., 2010; Ewers & Didham, 2006; Henle et al., 2004). Identifying barrier types that simultaneously affect multiple native species should facilitate the design of proper management strategies (Frank). Yet, increased connectivity potentially facilitates the spread of invasive species, which is a highly undesirable spin‐off of restoring natural connectivity in riverine systems (Terêncio et al., 2021). Genetic diversity of populations in the introduced range of non‐native species is generally lower compared to populations in the species' native range due to founder effects and range expansion (such as the associated allele surfing) (Demastes et al., 2019; Facon et al., 2006; Hardouin et al., 2018). However, this pattern might not be observed when individuals from multiple genetically diverse populations are introduced, increasing heterozygosity in the extended range (Bermond et al., 2012; Keller et al., 2014; Rosenthal et al., 2008).

In this study, we focus on genetic diversity and population genetic connectivity of three riverine fish species with contrasting dispersal capacity, life history strategy, ecology, and environmental tolerance. Three‐spined stickleback (*Gasterosteus aculeatus* Linnaeus 1758, Gasterosteidae) is a small (3–11 cm) eurytopic fish species, occupying various habitats (small ponds, lakes, riverine, and coastal habitats) and high pollution tolerance. It has a short generation time (1–2 years) and low to moderate dispersal capacities. Barriers such as watermills and weirs are significant drivers of genetic divergence (Raeymaekers et al., 2008, 2009). Stone loach (*Barbatula barbatula* Linnaeus 1758, Nemacheileidae) is a benthic and longer‐lived species (size: 10–20 cm, expected lifespan: 5–7 years) with low tolerance to environmental pollution (Wheeler, 1992). Previous population genetic studies identified strong IBD patterns driven by low dispersal abilities (Barluenga & Meyer, 2005; Fourtune et al., 2016; Knapen et al., 2009; Norén et al., 2018). Topmouth gudgeon (*Pseudorasbora parva* Temminck & Schlegel 1846; Cyprinidae) is a small (3–11 cm) non‐indigenous species, which can carry the highly infectious unicellular *Sphaerothecum destruens* parasite (Gozlan et al., 2010; Spikmans et al., 2020). The species is native to East‐Asia but has rapidly dispersed throughout Europe since its accidental introduction in Eastern Europe in the 1960s. The rapid dispersal is facilitated by a strong plasticity in life history traits, short generation time (sexually mature at the age of one year, lifespan up to five years), high reproductive effort, and wide environmental tolerance (Beyer et al., 2007; Britton et al., 2007; Pindera et al., 2005). Previous genetic studies have focused on large scale genetic patterns and suggested increased genetic diversity in its invasive range (Baltazar‐Soares et al., 2020; Brazier et al., 2022; Hardouin et al., 2018; Simon et al., 2011, Simon, 2012), suggesting multiple introduction sources (Baltazar‐Soares et al., 2020; Brazier et al., 2022). To date, not any study has focused on comparing patterns of genetic diversity and divergence of topmouth gudgeon on a local scale to native species. Such comparison might provide essential information on invasion success.

Our goal was to investigate (dis‐)similarity in genetic patterns between two native and an invasive species, and to determine the relative importance of natural (i.e. network centrality, downstream and upstream distance) and anthropogenic (i.e. migration barriers) factors underlying genetic diversity and connectivity. Based on the comparison among three co‐occurring fish species, we provide recommendations to reconcile the preservation of genetic diversity.

## 2 METHODS

### 2.1 Study area and sampling

**FIGURE 1:**
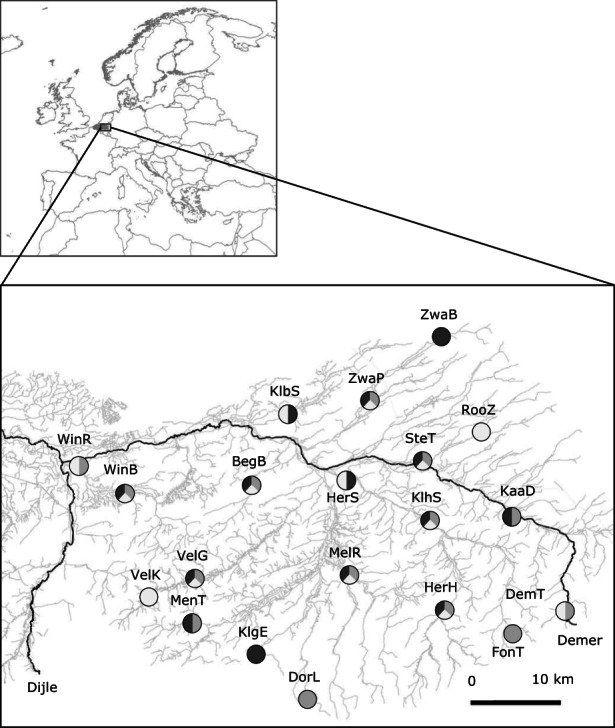
Overview of the 20 sampling locations (Demer basin, Flanders, Belgium). The black lines represent the main rivers (Left = Dijle, right = Demer). See Table 1 for site codes and geographic coordinates. Colours refer to the three species (three‐spined stickleback = grey, stone loach = dark grey, topmouth gudgeon = white).

Fish were sampled during autumn 2017 following a standardized electrofishing protocol under permission of the Agency of Nature and Forest (ANB) of the Flemish Community (Belgium). Fishing covered a river stretch of 100 m at each of the 20 locations in the Demer basin in Flanders (Figure 1). All fish caught were identified to species level and counted. Up to 30 individuals of stone loach, three‐spined stickleback, and topmouth gudgeon were collected, euthanized using MS222 following directions of the KU Leuven Animal Ethics Committee, and stored individually at −20°C. Fin clips were collected from each individual and stored in 70% ethanol. Surplus individuals of these species and all other fish were released on site. A total of 14 populations for each species were collected and included in this study. All species were present at eight locations. Two out of three species were present at six locations, and six locations included only one out of three species, resulting in a total of 20 locations included in this study (Figure 1, Table 1).

**TABLE 1:** Details of the sampling locations, including site code, river, longitude and latitude, average observed (*H*_*O*_) and expected (*H*_*e*_) heterozygosity, allelic richness (AR), and inbreeding coefficient (*F*_IS_) per sampling location and per species

Code	River	Location	Latitude	Longitude	Three‐spined stickleback				Stone loach				Topmouth gudgeon			
					*H*_*o*_	*H*_*e*_	AR	*F*_IS_	*H*_*o*_	*H*_*e*_	AR	*F*_*IS*_	*H*_*o*_	*H*_*e*_	AR	*F*_*IS*_
WinB	Winge	Blauwmolen	50.9386	4.8014	0.103	0.107	1.340	0.032	0.149	0.129	1.451	−0.109	0.156	0.150	1.545	−0.024
BegB	Begijnenbeek	Bekkevoort	50.9494	5.0029	0.104	0.104	1.322	0.003	0.143	0.131	1.461	−0.061	0.145	0.145	1.522	−0.002
ZwaP	Zwarte beek	Paal	51.0288	5.1921	0.095	0.092	1.267	−0.027	0.140	0.134	1.516	−0.023	0.154	0.157	1.615	0.019
SteT	Steenlaak	Tiewinkel	50.9651	5.2341	0.113	0.113	1.367	0.002	0.151	0.138	1.542	−0.060	0.151	0.150	1.591	0.000
MelR	Melsterbeek	Runkelen	50.8565	5.1596	0.118	0.113	1.374	−0.029	0.151	0.138	1.534	−0.065	0.147	0.147	1.545	0.003
KlhS	Kleine Herk	Stevoort	50.9178	5.2506	0.114	0.115	1.376	0.006	0.140	0.137	1.521	−0.014	0.137	0.147	1.566	0.047
VelG	Velpe	Glabbeek	50.8563	4.9501	0.105	0.109	1.346	0.023	0.132	0.124	1.429	−0.053	0.142	0.144	1.554	0.014
HerH	Herk	Hoepertingen	50.8071	5.2973	0.107	0.108	1.329	0.011	0.124	0.120	1.389	−0.024	0.120	0.128	1.414	0.051
KaaD	Kaatsbeek	Diepenbeek	50.9177	5.4153	0.115	0.116	1.379	0.005	0.117	0.110	1.350	−0.046	–	–	–	–
MenT	Mene	Tienen	50.8030	4.9254	0.106	0.106	1.335	0.002	0.139	0.134	1.541	−0.027	–	–	–	–
DemT	Demer	Tongeren	50.8163	5.5045	0.076	0.074	1.206	−0.018	–	–	–	–	0.139	0.137	1.467	−0.013
WinR	Winge	Rotselaar	50.9629	4.7200	0.113	0.112	1.364	−0.002	–	–	–	–	0.136	0.150	1.564	0.067
KlbS	Kleine beek	Schaffen	51.0108	5.0483	–	–	–	–	0.142	0.139	1.552	−0.023	0.148	0.152	1.594	0.017
HerS	Herk	Schulen	50.9536	5.1318	–	–	–	–	0.111	0.135	1.506	0.142	0.135	0.150	1.580	0.079
DorL	Dormaalbeek	Landen	50.7368	5.0847	0.107	0.106	1.316	−0.002	–	–	–	–	–	–	–	–
FonT	Fonteinbeek	Tongeren	50.7962	5.4194	–	–	–	–	–	–	–	–	–	–	–	–
KlgE	Kleine Gete	Ezemaal	50.7751	5.0028	–	–	–	–	0.131	0.131	1.475	−0.002	–	–	–	–
ZwaB	Zwarte beek	Beringen	51.0919	5.3175	–	–	–	–	0.136	0.135	1.519	−0.010	–	–	–	–
RooZ	Roosterbeek	Kerkom	50.8454	4.8817	–	–	–	–	–	–	–	–	0.138	0.146	1.550	0.040
VelK	Velpe	Zonhoven	50.9879	5.3550	–	–	–	–	–	–	–	–	0.141	0.150	1.585	0.048

### 2.2 Molecular methods and DNA sequencing

A total of 840 specimens were genotyped at genome‐wide SNPs sourced by Genotyping‐by‐Sequencing (GBS), comprising 20 individuals per population of each of the three species. First, genomic DNA was extracted from stored fin clips using a salt precipitation protocol adapted from Cruz et al. (2017). Next, DNA quality and quantity were assessed. DNA quantity was determined using Quant‐iT PicoGreen dsDNA kit (Thermo Fisher Scientific Inc.) according to the manufacturer's instructions. Quality was checked using agarose gel electrophoresis after diluting the samples to a concentration of 10 ng/μl. Individuals with low quality and/or quantity were discarded and replaced with another individual.

The final step included the construction of a GBS library following the methods described in Elshire et al. (2011), with modifications as described in Christiansen et al. (2021). Three pools of 96 individual libraries were prepared per species. Each pool included four control individuals. The choice of enzyme, together with the final size selection step, was based on simulated in silico genome digestions using SimRAD v0.96 (Christiansen et al., 2021; Lepais & Weir, 2014). For three‐spined stickleback, we used the reference genome (VDFJ00000000.1; Berner et al., 2019) for in silico digestions. Because there is no reference genome for topmouth gudgeon and stone loach, we simulated one using the sim.DNAseq function of the SimRAD package. This function randomly generates a DNA sequence (‘reference genome’) of a specific length and GC content. We conservatively used 1662.6 Mb and 528.12 Mb bp length with 40% and 41% GC content to generate in silico ‘reference genomes’ for topmouth gudgeon and stone loach, respectively. These length and GC values were inspired by genomic information from related species. After in silico optimization, the same laboratory protocol was used for the three species. In brief, individual DNA samples were digested using restriction enzyme *ApeKI* for 2 h at 75°C. Both a unique (5–7 bp) and common barcode were ligated to the digested DNA at 22°C for 60 min, followed by 30 min at 65°C. All samples were purified using CleanPCR beads before and after PCR amplification. The concentration of each sample was checked using the Quant‐iT PicoGreen dsDNA kit (Thermo Fisher Scientific Inc.). Based on the concentration, all samples from one pool were combined so that 10 ng from each individual sample was added. Each pooled library was size selected for fragments with a size ranging from 240 to 340 bp (excluding adapters) using a BluePippin (Sage Science). A more detailed protocol is added to the Supplements. Libraries were sequenced 100 bp paired end on a Illumina HiSeq2500 platform at Macrogen, Inc.

### 2.3 Bioinformatic analysis

Read quality of the raw sequencing files was assessed using FastQC software v0.11.9 (Andrews, 2014). The raw data was demultiplexed using the process_radtags module from Stacks v2.54 (Catchen et al., 2011, 2013; Rochette et al., 2019). During demultiplexing, reads with an uncalled base and low‐quality scores (Phred <10) were removed. Sequences containing one barcode or RAD‐Tag mismatch were rescued. Next, we followed the reference‐based pipeline of Stacks for three‐spined stickleback and the de novo pipeline for stone loach and topmouth gudgeon following established recommendations (Paris et al., 2017; Rochette et al., 2019; Rochette & Catchen, 2017).

The retained reads of three‐spined stickleback were mapped against the reference genome (VDFJ00000000.1; Berner et al., 2019). The reference genome was first indexed and GBS sequences were aligned using bowtie2 v2.4.1 (Langmead & Salzberg, 2012). Alignment rate varied between 52.45 and 96.63%. Next, SNPs were called using the gstacks module from Stacks with default settings. Loci were subsequently filtered using the populations module from Stacks. Loci present in less than ten populations were discarded. Similarly, a locus was only processed when present in at least 75% of the individuals per population.

For stone loach and topmouth gudgeon, SNPs were called using the *denovo_map* pipeline from Stacks. Various parameter combinations were screened (Paris et al., 2017; Rochette & Catchen, 2017). Based on the results, we selected a minimum coverage of three reads per stack (*m* = 3) and a maximum number of four base pair differences between stacks (*M* = 4) and between catalogue loci (*n* = 4). A locus catalogue was built, based on a subset of samples, before mapping all samples to this catalogue.

Additional SNP filtering, using the VCF file produced by Stacks, was conducted in R v4.0.2 with the package Radiator v1.28.1 (Table S1; Gosselin et al., 2020), for all three species. We first removed duplicated, and non‐common markers. Next, individuals and markers with missingness above 20% were removed. Markers and individuals with heterozygosity between 0.01 and 0.5, minor allele count of 3, and coverage between 10 and 100 were included. Finally, we accounted for short distance linkage disequilibrium, by retaining only one SNP per locus, and removed SNPs not following Hardy–Weinberg equilibrium. We removed the three‐spined stickleback samples of the Fonteinbeek because only six individuals remained after filtering.

### 2.4 Spatial data

Spatial (waterway) distances were calculated using the Network Analyst toolbox in ArcGIS v10.8.1 (ESRI, Belgium). Upstream distance was defined as the maximal upstream distance from the sampling site and downstream distance was defined as the distance to the Dijle‐Demer river confluence (50.96867 N; 4.6928 E). Network centrality was calculated as the average waterway distance of a sampling location to all other locations. Pairwise and downstream numbers of barriers between the sampling locations were counted, based on a data set of the Flemish Environment Agency (VMM) that keeps track of current and historic migration barriers, and divided into six categories: watermills, weirs, tunnels, sluices, riverbed obstructions, and others.

### 2.5 Statistical analysis

Statistical analyses were implemented in R v4.0.2 (R Core Team, 2020). All analyses were run for all 14 sampling locations and all three species, and for the eight locations where all three species co‐occurred.

#### 2.5.1 Population genetic structure

Genetic diversity was calculated as observed (*H*_*O*_) and expected (*H*_*E*_) heterozygosity per population using the R package DiveRsity v1.9.90 (Keenan et al., 2013). *F*_IS_ (1 − [*H*_*O*_/*H*_*E*_]) was calculated for each population using the DiveRsity package. Effective population size (*N*_*e*_) was calculated for each population with the linkage disequilibrium method (Hill, 1981; Waples, 2006) using a minimum allele frequency cutoff of 0.05 and a random mating model with NeEstimator v2.1 (Do et al., 2014). Next, 95% confidence intervals using the jackknife option were calculated for each population. Genetic differentiation was calculated as global and pairwise *F*_*ST*_ using the R package Hierfstat v0.5.7 (Goudet, 2005) and the DiveRsity package, respectively (Keenan et al., 2013). Genetic differentiation was visualized using principal component analysis (PCA; Adegenet v2.1.3; Jombart, 2008) and Structure software v2.3.4 (Falush et al., 2003). Structure was run three times with the R package ParallelStructure v1.0 (Besnier & Glover, 2013) using an admixture model without population priors (burn‐in = 10,000; iterations = 100,000). The number of clusters *K* was set to range from 2 to 14. The optimal value of *K* was evaluated based on *Delta K* and log likelihood using Structure Harvester v0.6.94 (Earl & vonHoldt, 2012).

#### 2.5.2 Spatial patterns of genetic diversity

The spatial distribution of observed and expected heterozygosity was evaluated for each species using a linear model with network centrality, upstream distance (distance of a sampling location to the most upstream part of the river), downstream distance (distance to the Dijle‐Demer confluence), and downstream number of barriers as explanatory variables. Collinear variables were removed (|*r*| > 0.6; Figure S1; Dormann et al., 2013) and the best model was selected using the Akaike Information Criterion (AIC = −2[log‐likelihood] + 2 *K*, with *K* being the number of model parameters). Stepwise model selection (both backward and forward) was performed using the stepAIC function in the R package MASS v7.3–54 (Venables & Ripley, 2013). The final model was the model with the lowest AIC.

#### 2.5.3 Spatial patterns of genetic differentiation

IBD was evaluated based on the correlation between pairwise *F*_*ST*_ values and pairwise waterway distances. Statistical significance was assessed using a simple Mantel test in ade4 v2.1.3 (Jombart, 2008). To identify the impact of barriers on genetic differentiation, we first performed simple and partial (to account for the correlation with waterway distance) Mantel tests to calculate the correlation between each of the six barrier categories and pairwise *F*_ST_ using ade4 v2.1.3 Adegenet v2.1.3 (Jombart, 2008).

#### 2.5.4 Redundancy analysis

We estimated the relative importance of natural and anthropogenic spatial parameters on genetic structure of each species with redundancy analysis (RDA) using the R package vegan v2.5–7 (Oksanen et al., 2013). Allele frequencies were Hellinger transformed and principal components with a cumulative variance of more than 75% were used for further analysis (three‐spined stickleback: 130 PCs, stone loach: 158 PCs, topmouth gudgeon: 157 PCs). For each species, we performed two separate RDAs. One RDA included spatial parameters. Next to network centrality, upstream and downstream distance, principal coordinates of neighbour matrices (PCNM) were calculated based on pairwise waterway distances. The second RDA included the downstream number of barriers (watermills, weirs, tunnels, sluices, riverbed obstructions). Collinear variables were removed prior to analysis (|*r*| > 0.6, Figure 1, Dormann et al., 2013) and the variance inflation factor (VIF) was calculated to ensure the absence of multicollinearity in the final model. Variables included in the final models were selected using stepwise forward and backward model selection based on adjusted R^2^ and P‐values using the ordiR2step function in the vegan R package (Oksanen et al., 2013).

## 3 RESULTS

### 3.1 DNA sequencing quality and filtering

A total of 1,297,011,662, 1,245,943,066, and 1,423,660,372 reads were generated from three species‐specific pooled GBS libraries of three‐spined stickleback, stone loach, and topmouth gudgeon respectively. After demultiplexing, quality trimming, and running the Stacks pipeline (reference based for three‐spined stickleback, de novo for stone loach and topmouth gudgeon), we retained 245,890, 114,819, and 258,817 variant sites in 286, 287, and 280 individuals, respectively. After filtering, the final data sets included 17,411 SNPs in 236 three‐spined stickleback, 17,407 SNPs in 255 stone loach, and 23,401 SNPs in 249 topmouth gudgeon (Table S1).

### 3.2 Population genetic diversity and structure

Mean observed heterozygosity (*H*_*O*_) ranged from 0.076 (Demer Tongeren) to 0.118 (Melsterbeek Runkelen) in three‐spined stickleback, from 0.117 (Kaatsbeek Diepenbeek) to 0.151 (Melsterbeek Runkelen) in stone loach, and from 0.120 (Herk Hoepertingen) to 0.156 (Winge Blauwmolen) in topmouth gudgeon (Table 1). Mean expected heterozygosity (*H*_*E*_) exceeded *H*_*O*_ at five sampling sites of three‐spined stickleback. In stone loach, *H*_*E*_ was consistently lower than *H*_*O*_. In topmouth gudgeon, *H*_*E*_ was lower than *H*_*O*_ at three sampling sites. Across the eight shared sampling locations, observed heterozygosity was correlated between stone loach and topmouth gudgeon (stone loach – topmouth gudgeon: Pearson *r* = 0.824, *p* = 0.018; three‐spined stickleback – stone loach: Pearson *r* = 0.302, *p* = 0.467; three‐spined stickleback ‐ topmouth gudgeon: Pearson *r* = −0.220, *P* = 0.601). Effective population size ranged from 19.1 (Demer Tongeren) to 392.1 (Mene Tienen) in three‐spined stickleback (average *Ne* = 98.5); from 130.2 (Begijnenbeek Bekkevoort) to infinite (Zwarte Beek Beringen) in stone loach (average *Ne* = 679.2); and from 135.5 (Begijnenbeek Bekkevoort) to 518.1 (Kleine Beek Schaffen) in topmouth gudgeon (average *Ne* = 279.84) (Table 2).

**TABLE 2:** Effective population size (*N*_*e*_) per population, calculated using the Linkage Disequilibrium method (Do et al., 2014) and 95% confidence intervals. Sampling codes are available in Table 1.

Code	Three‐spined stickleback	Stone loach	Topmouth gudgeon
WinB	68.9 (37.7; 323.3)	187.3 (48.1; ∞)	216 (62.4; ∞)
BegB	63.2 (32.2; 405.1)	130.2 (37.1; ∞)	135.5 (62.6; ∞)
ZwaP	124.2 (35.1; ∞)	136 (31.9; ∞)	364.3 (92.7; ∞)
SteT	116.8 (44.7; ∞)	503.6 (70.1; ∞)	208 (53.0; ∞)
MelR	121.9 (46.3; ∞)	234.2 (58.2; ∞)	284.5 (76.2; ∞)
KlhS	114.3 (50.7; ∞)	∞ (5932.5; ∞)	291.4 (103.1; ∞)
VelG	47.5 (21.0; ∞)	108.4 (47.8; ∞)	441.5 (127.5; ∞)
HerH	51.6 (28.9; 163.4)	963.8 (96.9; ∞)	129.8 (59.4; ∞)
KaaD	52.4 (29.0; 175.8)	368.6 (95.9; ∞)	–
MenT	392.1 (94.5; ∞)	656.2 (198.8; ∞)	–
DemT	19.1 (11.7; 38.6)	–	138.0 (48.1; ∞)
WinR	42.3 (19.5; 600.5)	–	140.8 (34.5; ∞)
KlbS	–	3236.3 (151.1; ∞)	518.1 (67.7; ∞)
HerS	–	373.3 (59.2; ∞)	256.2 (77.5; ∞)
DorL	65.7 (39.0; 175.1)	–	–
FonT	–	–	–
KlgE	–	–	–
ZwaB	–	1252.5 (98.8; ∞)	301.3 (106.5; ∞)
RooZ	–	∞ (251.5; ∞)	492.3 (126.7; ∞)
VelK	–	–	–

Overall *F*_*IS*_ was highest in populations of topmouth gudgeon (average = 0.063) varying between −0.024 and 0.079, followed by three‐spined stickleback (average = 0.044, range = −0.029–0.043) and stone loach (average = −0.008, range = −0.109–0.142; Table 1). We observed the strongest population genetic differentiation in three‐spined stickleback (global *F*_*ST*_ = 0.117), followed by stone loach (global *F*_*ST*_ = 0.081) and topmouth gudgeon (global *F*_*ST*_ = 0.038). Pairwise *F*_*ST*_ ranged from 0.047 to 0.329 in three‐spined stickleback, and all pairwise comparisons were significant (Table S2). Pairwise *F*_*ST*_ of stone loach ranged from 0 to 0.222 with ten non‐significant estimates (Table S3). In topmouth gudgeon, 14 pairwise comparisons were not significant (range = 0–0.137, Table S4). At the eight shared sampling locations, pairwise comparisons were significantly correlated between stone loach and topmouth gudgeon (Mantel *r* = 0.750, *p* = 0.019), but not with three‐spined stickleback (three‐spined stickleback – stone loach: Mantel *r* = 0.220, *p* = 0.134; three‐spined stickleback – topmouth gudgeon: Mantel *r* = 0.159, *p* = 0.209).

**FIGURE 2:**
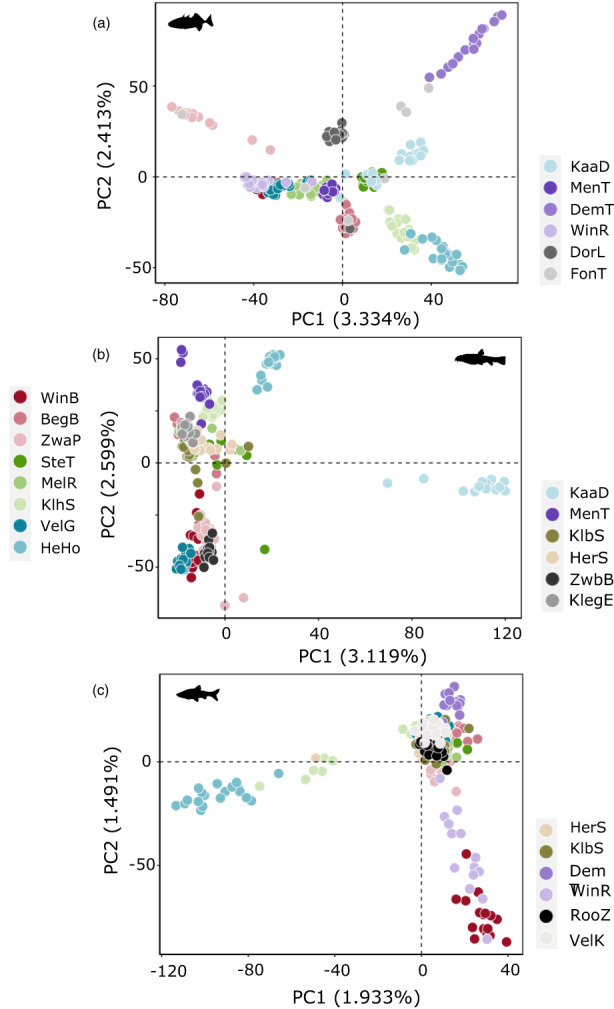
Principal Component Analysis biplot of the SNP genotypes per site in three‐spined stickleback (a), stone loach (b), and topmouth gudgeon (c). Colours refer to the sampling sites; for site abbreviations see Table 1.

**FIGURE 3:**
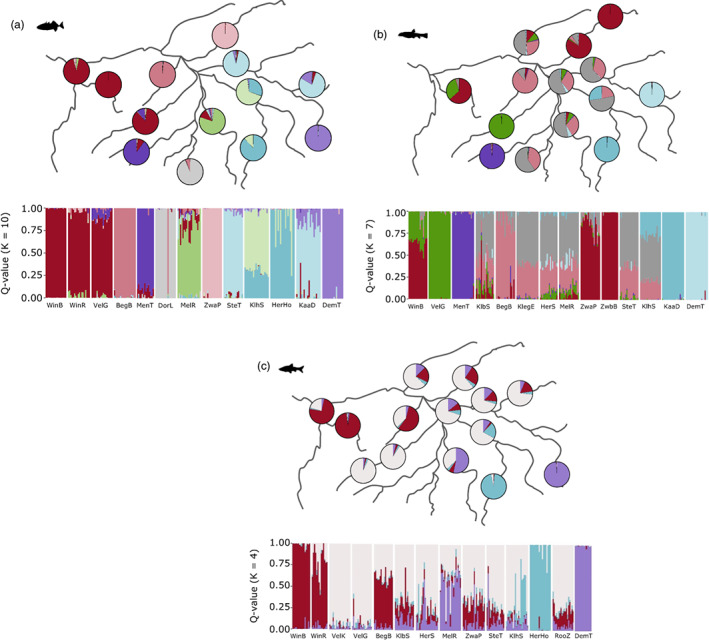
Structure pie chart with average assignment to each of the clusters per population (above) and bar chart with assignment to each of the clusters per individual (below) for three‐spined stickleback (a), stone loach (b), and topmouth gudgeon (c). For site abbreviations see Table 1. The order of the populations in the bar chart shows the populations from west to east.

Principal component analysis revealed clear differentiation among populations of three‐spined stickleback, stone loach, and topmouth gudgeon (Figure 2). Furthermore, patterns differed between species and the strongest population differentiation was observed in three‐spined stickleback, while in topmouth gudgeon only three groups were separated by the first two PC axes. In stone loach, the first PC axis clearly differentiated Kaatsbeek Diepenbeek and Herk Hoepertingen, while most other locations were separated on the second PC axis. Clustering analysis identified ten groups of three‐spined stickleback, seven groups of stone loach and four groups of topmouth gudgeon (Figure 3). General patterns differed between species. However, some locations showed distinct populations for all three species (e.g. the upstream sites Herk Hoepertingen, Mene Tienen, Kaatsbeek Diepenbeek, and Demer Tongeren).

### 3.3 Riverscape genomics

#### 3.3.1 Spatial patterns of genetic diversity and differentiation

**FIGURE 4:**
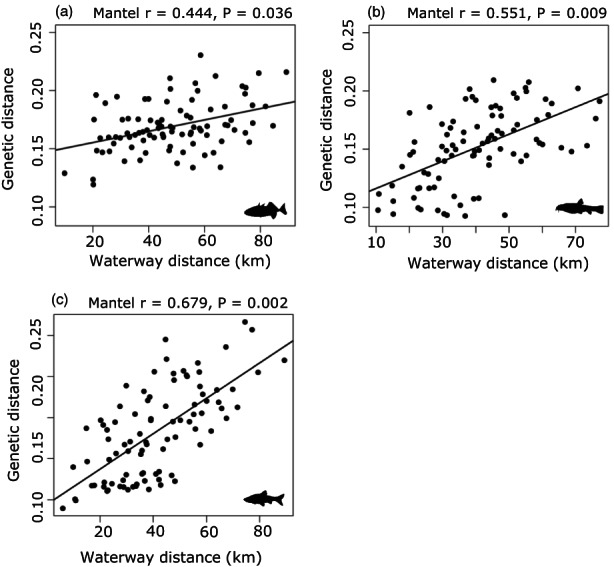
Isolation‐By‐Distance plot based on pairwise waterway distances and genetic distance (*F*_ST_/1 − *F*_ST_) of three‐spined stickleback (a), stone loach (b), and topmouth gudgeon (c).

The observed heterozygosity of the three‐spined stickleback populations was not significantly correlated to any spatial variable (network centrality: *F*_1,11_ = 3.703, *p* = 0.078; downstream distance: *F*_1,11_ = 1.739, *p* = 0.078). In stone loach, upstream (*F*_1,12_ = 3.336, *p* = 0.095) and downstream distances (*F*_1,12_ = 4.433, *p* = 0.059) were included in the final model but were not associated with observed heterozygosity when including all locations. In topmouth gudgeon, total number of downstream barriers (*F*_1,11_ = 10.178, *p* = 0.010) was negatively correlated with observed heterozygosity. Upstream distance (*F*_1,11_ = 4.559, *p* = 0.059) and network centrality (*F*_1,11_ = 2.133, *p* = 0.175) were also included in the final model but did not significantly affect observed heterozygosity. We observed IBD (Figure 4) in three‐spined stickleback (Mantel *r* = 0.444, *p* = 0.008), stone loach (Mantel *r* = 0.551, *p* = 0.009), and topmouth gudgeon (Mantel *r* = 0.679, *p* = 0.002). When including the eight shared locations, overall patterns remained similar (Figure S2) with the strongest IBD pattern observed in topmouth gudgeon (Mantel *r* = 0.642, *p* = 0.041), followed by stone loach (Mantel *r* = 0.574, *p* = 0.048). No IBD was observed in three‐spined stickleback across these eight locations (Mantel *r* = 0.164, *p* = 0.221).

Population differentiation was correlated with pairwise number of barriers, riverbed obstructions, watermills, and tunnels in the topmouth gudgeon populations, before and after correcting for waterway distances (Table 3). Population differentiation of three‐spined stickleback and stone loach was not correlated with any barrier type after correcting for waterway distances. Observed patterns were similar when only including the eight overlapping sampling locations (Table S5).

**TABLE 3:** Simple and partial Mantel tests correlating genetic distance (*F*_ST_/1 − *F*_ST_) and distance based on pairwise number of barriers in three‐spined stickleback, stone loach, and topmouth gudgeon.

		Three‐spined stickleback		Stone loach		Topmouth gudgeon	
		Simple	Partial	Simple	Partial	Simple	Partial
Riverbed obstructions	*r*	0.035	−0.105	0.244	0.083	**0.675**	**0.616**
	*p*	0.401	0.623	0.172	0.361	**0.006**	**0.015**
Weirs	*r*	0.067	−0.079	0.204	0.136	0.422	0.440
	*p*	0.341	0.595	0.200	0.291	0.063	0.073
Watermills	*r*	0.296	0.120	**0.440**	0.298	**0.759**	**0.594**
	*p*	0.104	0.301	**0.043**	0.135	**0.002**	**0.016**
Tunnels	*r*	−0.107	−0.112	−0.132	−0.197	0.390	**0.532**
	*p*	0.621	0.666	0.681	0.757	0.091	**0.015**
Sluices	*r*	−0.002	−0.199	0.031	−0.317	0.284	−0.127
	*p*	0.390	0.829	0.401	0.932	0.108	0.685
Others	*r*	0.346	0.218	−0.203	−0.484	0.366	−0.052
	*p*	0.078	0.180	0.806	0.992	0.068	0.549
Total	*r*	0.182	−0.055	0.263	0.010	**0.845**	**0.707**
	*p*	0.199	0.571	0.130	0.465	**0.001**	**0.001**

*Note*: Significant values are in bold.

#### 3.3.2 RDA

**FIGURE 5:**
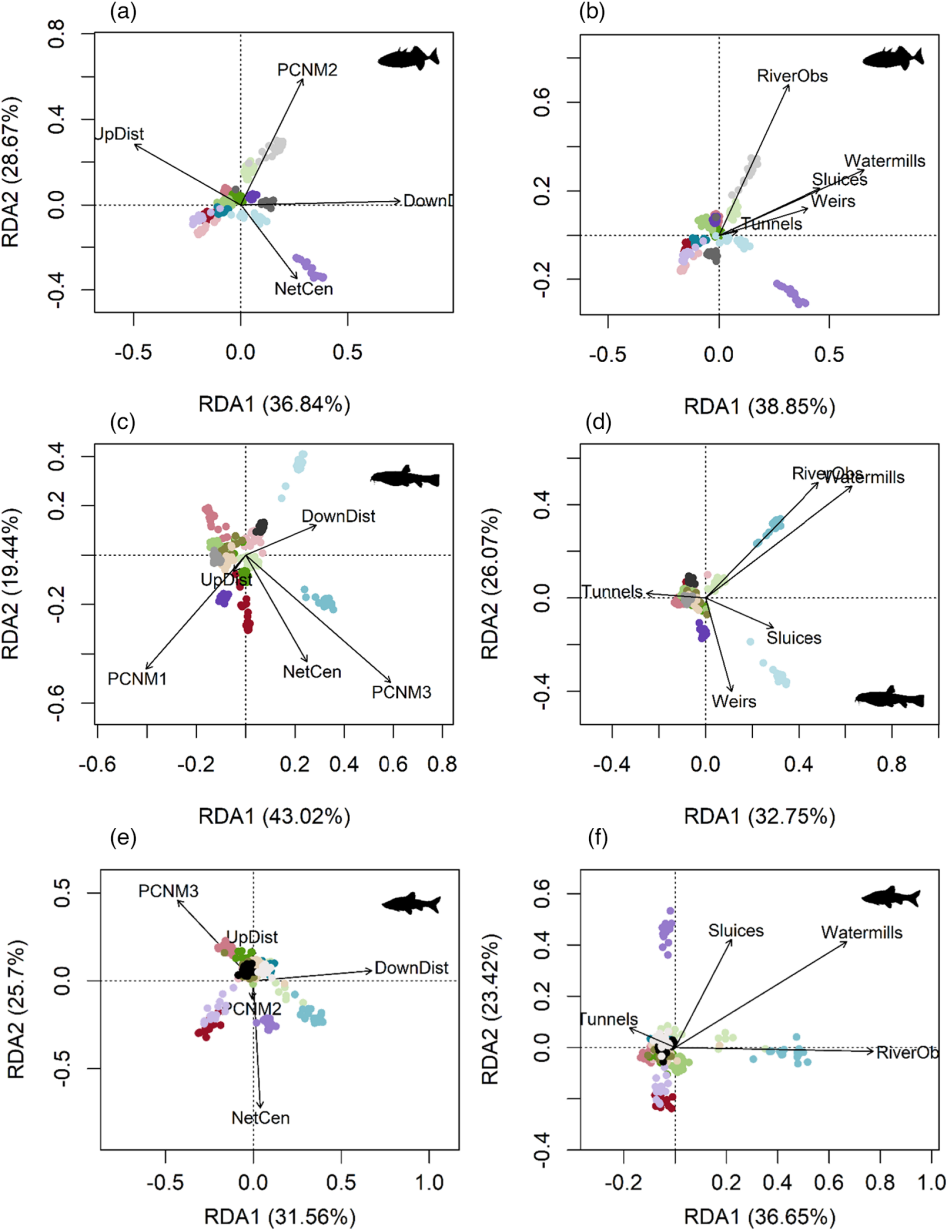
Redundancy analysis biplot linking the natural (a, c, e) and anthropogenic (b, d, f) spatial data sets to the genetic composition of three‐spined stickleback (a, b), stone loach (c, d), and topmouth gudgeon (e, f). Dots represent individuals and colours match with the populations (see Figure 2 for the colour legend and Table 1 for the site abbreviations).

The natural and anthropogenic spatial variables significantly predicted genetic composition of three‐spined stickleback, stone loach, and topmouth gudgeon (Figure 5, Table 4, Table S6). However, the proportion of variation explained and the importance of the various parameters differed between species. Both data sets explained most variation in the genetic composition of three‐spined stickleback, with the highest contribution from the barrier data set (barriers: adjusted *R* ^*2*^ = 0.141; natural spatial variables: adjusted *R* ^*2*^ = 0.111). Downstream distance was the most important variable of the spatial data set (Figure 5a), while the presence of watermills was most important in the barrier data set (Figure 5b).

**TABLE 4:** Variation partitioning results including adjusted *R* ^2^, *F*‐value and *p*‐value for the natural and anthropogenic spatial data sets

	Three‐spined stickleback					Stone loach					Topmouth gudgeon				
	DF	Variance	*F*	*p*	Adjusted *R* ^2^	DF	Variance	*F*	*p*	Adjusted *R* ^2^	DF	Variance	*F*	*p*	Adjusted *R* ^2^
Total effect space	4	0.007	8.126	0.001	0.111	5	0.006	5.539	0.001	0.081	5	0.004	3.246	0.001	0.043
Unique effect space	4	0.005	6.747	0.001	0.079	5	0.004	4.285	0.001	0.054		0.499	1.971	0.001	0.022
Network centrality	1	0.002	7.895	0.001	–	1	0.008	3.635	0.001		1	0.001	3.288	0.001	
Upstream distance	1	0.001	5.946	0.001	–	1	0.001	2.063	0.001		1	0.001	2.544	0.001	
Downstream distance	1	0.002	7.842	0.001	–	1	0.001	5.537	0.001		1	0.001	3.909	0.001	
PCNM1	–	–	–	–	–	1	0.002	7.673	0.001		–	–	–	–	
PCNM2	1	0.002	8.477	0.001	–	–	–	–	–		1	0.001	2.561	0.001	
PCNM3	–	–	–	–	–	1	0.001	4.018	0.001		1	0.001	2.176	0.001	
Total effect barriers	5	0.009	8.486	0.001	0.141	5	0.008	7.403	0.001	0.110	4	0.005	4.836	0.001	0.058
Unique effect barriers	5	0.007	7.157	0.001	0.106	5	0.006	6.074	0.001	0.084	4	0.003	3.258	0.001	0.040
Riverbed obstructions	1	0.002	10.319	0.001		1	0.002	9.170	0.001	1	1	0.001	5.016	0.001	
Weirs	1	0.001	6.000	0.001		1	0.002	8.849	0.001	–	–	–	–	–	
Watermills	1	0.003	12.271	0.001		1	0.002	9.862	0.001	1	1	0.001	4.312	0.001	
Tunnels	1	0.001	5.574	0.001		1	0.002	7.040	0.001	1	1	0.001	3.969	0.001	
Sluices	1	0.001	5.296	0.001		1	0.001	4.407	0.001	1	1	0.001	3.984	0.001	
Combined effect					0.019					0.027					0.022

In stone loach, the spatial data set explained most genetic variation (barriers: adjusted *R* ^*2*^ = 0.110; natural spatial variables: adjusted *R* ^*2*^ = 0.081). The most important variables were PCNM3 in the spatial data set (Figure 5c) and watermills in the barrier data set (Figure 5d). Overall, both data sets explained the least variation in topmouth gudgeon; the barrier data set was the most important contributor (barriers: adjusted *R* ^*2*^ = 0.058: natural spatial variables: adjusted *R* ^*2*^ = 0.043). Important variables included downstream distance, riverbed obstructions, and watermills. In all three species only a small portion of the variation was explained by the combined effect of barriers and space (Table 4). General patterns remained the same when only including the eight shared sampling locations (Figure S3; Table S6).

## 4 DISCUSSION

Understanding and comparing genetic responses of fishes to natural and anthropogenic spatial variation is important to ensure long‐term persistence of species, especially given the current rapid rate of environmental change (Dudgeon et al., 2006). Restoring connectivity may decrease population susceptibility to loss of genetic diversity, inbreeding and even extinction, but the simultaneous effect of barrier removal on the dispersal of invasive species remains to be ascertained (Hoffmeister et al., 2005). Overall, genetic structure differed between three common fishes inhabiting Flemish river systems. Populations of the invasive topmouth gudgeon were less differentiated compared to the native species, three‐spined stickleback and stone loach. Despite the lower levels of population differentiation and high effective population size, topmouth gudgeon appeared strongly affected by both natural spatial variation and river fragmentation.

### 4.1 Population genetic structure varies across species

Populations of topmouth gudgeon showed significant genetic differentiation, although population genetic structure was not as pronounced as in the two native species. Effective population size was higher in topmouth gudgeon than in three‐spined stickleback, but lower than in stone loach. Large effective population sizes have previously been observed in topmouth gudgeon in Western Europe (Brazier et al., 2022). These observations suggest high gene flow between populations of topmouth gudgeon with minor effects of dispersal distance and spatial barriers on the connectivity among populations. Alternatively, even if isolated, populations of topmouth gudgeon have not experienced sufficient mutation, selection, and genetic drift since introduction. Consequently, population structure in topmouth gudgeon may be more affected by recent colonization and founder effects (Szűcs et al., 2014). It is thought that the initial accidental introduction of topmouth gudgeon in Europe occurred in the early 1960s in the Black Sea basin, through co‐introduction with grass and silver carp eggs from the People's Republic of China. Several introductions occurred simultaneously in Hungary, Lithuania, Romania and Ukraine (Gozlan et al., 2010). Following initial introductions, primary long distance introduction pathways were the result of translocations in aquaculture and recreational fishing, with natural dispersal, angling and ornamental fish trade as secondary pathways for dispersal (Gozlan et al., 2010). Topmouth gudgeon was observed in Flanders for the first time in the early 1990s and populations continue to expand (Verreycken et al., 2007). Dispersal continues through live bait by anglers and river flooding, potentially concealing natural dispersal and responses to riverine features (Verreycken et al., 2007). Three‐spined stickleback and stone loach, on the other hand, have been established since the early Holocene in Western Europe (Barluenga & Meyer, 2005; Mäkinen & Merilä, 2008; Wheeler, 1992). Hence, their genetic population structure has been shaped by historic patterns of natural selection and connectivity.

Higher levels of heterozygosity were observed in topmouth gudgeon compared to the other species. It is generally hypothesized that invasive species are genetically less diverse due to a succession of genetic bottlenecks associated with founder effects (Allendorf & Lundquist, 2003; Hardouin et al., 2018). Yet, multiple studies report higher genetic diversity in the invasive range of topmouth gudgeon compared to its native range (Baltazar‐Soares et al., 2020; Brazier et al., 2022; Simon et al., 2011, 2014). The most recent analysis of the global population genetics of topmouth gudgeon (Brazier et al., 2022) indicates that the invasive populations found in Europe descend from native admixed population, thus potentially explaining the elevated heterozygosity, which might also increase these populations' fitness by heterosis (Keller et al., 2014; Roman & Darling, 2007; Rosenthal et al., 2008).

### 4.2 Genetic responses to natural river network structure

Population genetic structure clearly varied among the three species (Blanchet et al., 2010; Raeymaekers et al., 2017). Yet, factors driving these differences remain to be determined. All three species showed different levels of IBD. IBD was strongest for topmouth gudgeon and weakest for three‐spined stickleback. IBD is common in riverine fishes (e.g. Blanchet et al., 2010; Brauer et al., 2016; Hänfling et al., 2004) and has been reported in three‐spined stickleback (Raeymaekers et al., 2008) and stone loach (Barluenga & Meyer, 2005; Knapen et al., 2009). Variation in the strength of IBD has been attributed to variation in species‐specific dispersal ability (Blanchet et al., 2010). However, the dispersal ability of our focal species probably does not contribute much to the observed pattern, because both stone loach and the resident ecotype of three‐spined stickleback have limited dispersal capacities (Barluenga & Meyer, 2005; Raeymaekers et al., 2008). The differences in IBD patterns suggest that topmouth gudgeon is more affected by spatial processes following recent colonization, compared to native species. Moreover, local selection may have contributed more to population differentiation in native species. For instance, local pollution may be genotoxic, select for tolerant genotypes, cause local bottlenecks, or alter migration patterns (Calboli et al., 2021; Costa, 2021; Díez‐del‐Molino et al., 2018). The variation in IBD patterns may also be attributed to variation in effective population size. Only a weak pattern was observed in three‐spined stickleback. Interestingly, this is the species with the lowest effective population size, followed by topmouth gudgeon and stone loach. It is suggested that IBD patterns are strengthened in small populations (Leblois et al., 2006), which is not the case in this system. However, some studies indicate the oppposite (e.g. Cuveliers et al., 2011).

Remarkably, other commonly observed natural spatial patterns were weak and only marginally significant. Heterozygosity slightly increased in more downstream populations in three‐spined stickleback and stone loach (downstream increase in intraspecific genetic diversity – DIGD, e.g. Paz‐Vinas et al., 2015) and was correlated with network centrality in three‐spined stickleback. A DIGD pattern can be induced by downstream‐biased gene flow, reducing the downstream effects of genetic drift, and local selection (Cyr & Angers, 2011; Morrissey & De Kerckhove, 2009; Paz‐Vinas et al., 2013). Similarly, higher diversity in more central sites may be associated with a higher connectivity (Altermatt, 2013). This may suggest that other (local) processes influence population differentiation in this study system.

### 4.3 Genetic responses to anthropogenic river fragmentation

Natural spatial patterns may be obscured by the interruption of riverine connectivity. Man‐made barriers such as dams, weirs, and watermills are key drivers of genetic structure in many freshwater fishes (Blanchet et al., 2010; Junker et al., 2012; Raeymaekers et al., 2008; Wofford et al., 2005). Mantel tests, however, indicated that genetic differentiation was only affected by the pairwise number of watermills in stone loach, and riverbed obstructions, watermills, tunnels, and the total number of barriers in topmouth gudgeon, after correcting for distance. Unlike Raeymaekers et al. (2008), none of the barriers appeared to affect neutral genetic differentiation in three‐spined stickleback. Our results are supported by previous studies suggesting that responses to fragmentation are species‐specific (Blanchet et al., 2010; Prunier et al., 2018). Species‐specific variation in dispersal ability, body size, habitat specialization, and colonization history all contribute to IDB patterns (Blanchet et al., 2010; Prunier et al., 2018). Despite morphological and ecological differences, connectivity between populations of both native species is not measurably affected by barriers in our data. After the creation of a dispersal barrier, reduced gene flow might be masked by large effective population sizes for some time. Depending on the age of the barriers, this may be the case for stone loach, as estimates of population size were high, but not for three‐spined stickleback. Alternatively, both species might have greater dispersal capacities than previously thought. Observed patterns of IBD may reflect historical disturbance of gene flow, for instance, by poor water quality (Deflem et al., 2021). Nevertheless, the strong genetic responses of topmouth gudgeon to both natural and anthropogenic spatial variation may suggest that species with recent range expansions and colonization history are more affected by spatial processes, even when effective population sizes are high.

Interestingly, Raeymaekers et al. (2008) observed a strong influence of weirs and watermills on three‐spined stickleback populations, using microsatellite markers in the same geographic region. However, both data sets only partly overlapped and were sampled 15 years apart. Connectivity may have increased in the recent past due to a strong focus on the removal of migration barriers (VMM, 2020). Moreover, redundancy analyses revealed that the genetic composition of three‐spined stickleback and stone loach was better explained by the spatial variables than for the invasive topmouth gudgeon. Mantel tests may potentially be too conservative, masking the effect of barriers on both native species or as suggested, other factors influence population genetic differentiation in both native species. Mantel tests only reveal the influence of barriers on genetic differentiation, while redundancy analysis reveals the influence on genetic composition, which may still reflect the influence of barriers. The results may also be attributed to a recent increase in connectivity in three‐spined stickleback and stone loach in response to the EU Water Framework Directive, with still observable effects of past population fragmentation (Santos et al., 2013). Similarly, the low contribution of spatial variables in topmouth gudgeon may suggest that the genetic signature of the native range remains strong. Moreover, although we observe a significant effect, population differentiation in topmouth gudgeon remains low in comparison to both native species.

### 4.4 Management implications

Understanding the factors driving genetic structure is essential to identify effective species conservation and management action plans. Comparison of empirical patterns between species shows whether it is feasible to identify a single model species, representing the responses of other species in an entire community (Blanchet et al., 2010). Our results, however, indicate that conservation of genetic diversity requires species‐specific actions in an ecosystem context, as suggested earlier in the freshwater (Blanchet et al., 2010; Prunier et al., 2018; Raeymaekers et al., 2017) and marine (Reiss et al., 2009; Vandamme et al., 2021) literature.

Following the outcome of redundancy analysis, riverbed obstruction and watermills were identified as strong determinants of genetic structure in all three species. This is not unexpected as long standing fragmentation by watermills is widespread, has left asymmetric genetic signatures and is worrying (Prunier et al., 2020; Raeymaekers et al., 2009). Solutions to diminish the impact of watermills include fish stocking and translocation, barrier removal and the construction of fish passages (Blanchet et al., 2010; Giller & Malmqvist, 1998). The latter seems to be the only feasible solution, as watermills are an integral part of the local cultural heritage and unlikely to be removed (Raeymaekers et al., 2009). Many studies have indeed confirmed the efficiency of fish passages (e.g. Agostinho et al., 2002; Pelicice & Agostinho, 2008), although they might not fully compensate the negative impact of the barrier (Noonan et al., 2012). Riverbed obstructions, on the other hand, are not absolute barriers but may interrupt connectivity by changing flow regimes and the loss of natural habitat (Crooks & Kay, 2015). Additionally, our results might guide specific management actions in the Demer basin in Flanders. Currently, a large engineering project is implemented where the flood plain of the Demer is again integrated in water management (VMM, 2018). The Sigma Plan aims at reconnecting meanders and lowering the winter dyke to summer dyke level to increase water retention and improve water quality. This has proven successful in the first implementations of the Sigma Plan in the Scheldt river. The increased nature development of the Demer basin with upstream restauration of the inundation plain should facilitate flood risk management, improve ecosystem services and counter habitat fragmentation. For example, the low genetic diversity in some of the most upstream locations (e.g. Herk Hoepertingen and Demer Tongeren), partially attributed to their upstream location but also impacted by historical isolation, might benefit from increased connectivity.

However, several challenges accompany the removal of barriers when restoring a river axis. Removal changes habitat from an impoundment river system to an open river system, with associated changes in hydroregime (Noonan et al., 2012). In our case removal restores river connectivity of native species but at the same time may facilitate upstream colonization by non‐native species or populations (Rahel & McLaughlin, 2018). Indeed, dispersal of topmouth gudgeon is constrained by the presence of barriers, suggesting that removal will facilitate dispersal to more upstream sampling locations. This response is highly undesirable, given the strong negative impact of topmouth gudgeon and other invaders on native fish communities (Gozlan et al., 2010). Colonization of upstream habitats by topmouth gudgeon will potentially increase competition, especially with three‐spined stickleback (Gozlan et al., 2010; Rahel, 2013). A similar scenario is occuring with several highly invasive Ponto‐Caspian freshwater gobies in the Meuse and Scheldt basin (Huyse et al., 2015; Verreycken et al., 2007). Restorative measures should weigh the threat of upstream colonization of invasive species against the extinction risks or isolation of native species case‐by‐case (Crooks & Kay, 2015; Fausch et al., 2009). For example, genetic diversity is low in the upstream part of the Demer as a result of the large number of barriers. Removing these barriers and restoring connectivity might be the best option to increase genetic diversity. However, the high abundance of topmouth gudgeon downstream represents a challenge. Here, the principle should rule that removing fragmentation has priority over maintaining fragmentation to control a hazard, more specifically preventing access to non‐native species. In addition, topmouth gudgeon is an opportunistic species whose competitive advantage might be curtailed in healthy ecosystems. The Flemish government is currently slowly answering to the European Water Framework Directive through water treatment of household sewage, improved farming practices and river restoration (Deflem et al., 2021).

Most interestingly, we argue that redundancy analysis may help decision making in specific locations. For example, heterozygosity in stone loach is low, while genetic differentiation is high in Kaatsbeek Diepenbeek. We identified the number of watermills and riverbed obstructions as a driver of this effect, suggesting that the construction of fish ladders should be prioritized. Similarly, two central locations (Kleine Beek Schaffen and Herk Hoepertingen) harbour distinct populations of both stone loach and three‐spined stickleback. Both locations appear strongly affected by the downstream number of barriers, indicating a need for barrier removal.

## 5 CONCLUSION

Genetic patterns differed between two native and one invasive riverine fishes. Although barriers influenced the genetic composition of both native species, we observed a strong effect on genetic differentiation in topmouth gudgeon. Hence management measures should focus on restoring connectivity, while minimizing further dispersal of the invasive topmouth gudgeon through improved water and habitat quality.

## CONFLICT OF INTEREST

The authors declare no conflict of interest.

## Supporting information


Appendix S1

## Data Availability

The data that support the findings of this study are openly available in Dryad at https://doi.org/10.5061/dryad.s1rn8pkbx.
