# Supplementary material for: Contrasting population genetic responses to migration barriers in two native and an invasive freshwater fish
Source: Evol Appl. 2022 Nov 5;15(12):2010–27. doi: 10.1111/eva.13469 (PMC9753842; doi:10.1111/eva.13469)
Supplement: Supplementary file 1 — Appendix S1 [file EVA-15-2010-s001.docx]

**GBS protocol**

1/ Prepare 2 plates with 10µl of each DNA sample at a concentration of 10ng/µl

2/ Restriction enzyme digestion of 192 samples with PstI or ApeKI (NEB, New England Biolabs)

- Add 6 μL of adaptor (1/10 diluted) to each well with multipipet
- prepare mix for 220 samples
- NEBbuffer 3: 440 μL
- PstI or ApekI: 220 μL
- Water: 220 μL

Vortex, spin down and put on ice

- Distribute 110 μL of the mix in 8 well strip and add 4 μL to each well with multipipet
- TOTAL volume in each well: 20 μL
- For PstI: incubate 2h at 37°C, cool down to 10°C
- For ApeKI: incubate 2h at 75°C, cool down to 10°C

3/ Ligation (T4 ligase from NEB)

- Prepare mastermix for 220 samples in falcon tube
  - - 10x T4 DNA ligase buffer: 1100 μL
    - T4 ligase: 264 μL
    - water: 5236 μL
- Distribute in a clean plastic tray and add 30 μL to each well containing digested DNA with multipipet
- TOTAL volume in each well: 50 μL
- Incubate 1 hour at 22°C, followed by 30’ at 65°C

4/ Purification with CleanPCR beads (CleanNA; GC Biotech) to reduce costs of CleanPCR beads, only 25 μL will be purified

- Add 25 μL beads to new plate
- Add 25 μL of digestion/ligation mixture and pipet up and down to ensure proper mixing mix by 10 times up and down pipetting
- Incubate 5 min at room temperature
- Place plate on magnet for 5 min to separate beads from solution
- Remove 45 μL of the clear solution while the plate is still on the magnet. Discard solution. Avoid taking out any beads, leave ca 5 μL of the solution behind.
- Add 200 μL of 70% ethanol and wait 30s
- Remove 200 μL ethanol (beads are now attached much better to the wall)
- Add 200 μL of 70% ethanol, wait 30s
- Remove all supernatant (230 μL of ethanol). Check whether all ethanol is removed. Take 10 μL multipipet to doublecheck whether all wells are empty. Residual ethanol may interfere with downstream PCR
- Remove plate from magnet and add 40 μL elution buffer (eg from Qiagen kit) or pure water (Sigma)
- Mix by pipetting 10X up and down
- Incubate 5 minutes
- Put plate on magnet for 5 minutes to separate beads from solution
- transfer 35 μL to new plate (to be sure no beads are transferred, although they do not form a problem later on)

5/ PCR on separate samples

- Prepare master mix for 200 samples
  - NEB Q5 hotstart mastermix: 200 x 12.5µl
  - Water: 200 x 10.5µl
  - Primermix: 200 x 1µl (contains F and R primer, each at 5 μM)
- Distribute 24 μL of the mix and add 1 μL of cleaned Ligation product
- TOTAL volume: 25 μL
- Initial denaturation at 98°C for 30”, followed by 18 cycles of 10” at 98°C, 30” at 65°C and 30” at

72°C. Final elongation 5’ at 72°C.

6/ Purification with CleanPCR beads

- purify PCR product as in step 3 but add only 20µl of beads to 25µl PCR reaction
- follow protocol in step 3
- the final elution volume is 30µl and 25µl is transferred to a new tube

7/ Quantification

PICOGREEN

8/ Pooling of the samples

Depending on the lowest concentration take 5 or 10 ng from each sample and quantify the pooled sample with picogreen.

**Suppl. Table 1a** Overview of the bio-informatics and SNP filtering for three-spined stickleback.

|  |  | **Library 1** | **Library 2** | **Library 3** |
| --- | --- | --- | --- | --- |
| N raw reads | - | 399 216 448 | 451 881 286 | 445 913 928 |
| N reads - Demultiplexing | Barcode Not Found | 5 673 622 (1.4%) | 1 427 554 (0.3%) | 1 514 604 (0.3%) |
|  | Low Quality | 1 857 936 (0.5%) | 2 392 615 (0.5%) | 2 186 746 (0.5%) |
|  | RAD Cutsite Not Found | 25 504 179 (1.6%) | 4 622 767 (1.0%) | 7 315 231 (1.6%) |
|  | Retained | 366 180 711 (97.5%) | 443 438 350 (98.1%) | 434 897 347 (97.5%) |
|  | N Individuals | 96 | 95 | 96 |
| N Loci - Stacks | Reference based | 4 540 943 | | |
|  | Denovo |  | | |
|  | Populations | 245 890 (286 individuals) | | |
| SNP filtering | Removing duplicated, | 82 657 (286 individuals) | | |
|  | monomorphic and non-common markers |  |  |  |
|  | Removing individuals with missingness | 82 657 (257 individuals) | | |
|  | above 20% |  |  |  |
|  | Heterozygosity markers 0.01-0.5 | 82 657 (257 individuals) | | |
|  | MAC 3 | 310 | | |
|  | Coverage 10-100 | 35 512 (257 individuals) | | |
|  | Marker missing proportion 20% | 35 512 (257 individuals) | | |
|  | SNP read position: based on outliers | 35 510 (257 individuals) | | |
|  | SNP per locus: based on outliers | 34 511 (257 individuals) | | |
|  | Short LD: based on MAC | 17 419 (257 individuals) | | |
|  | Heterozygosity individuals 0.01-0.5 | 17 419 (257 individuals) | | |
|  | Duplicate samples: 0.25 | 17 419 (236 individuals) | | |
|  | HWE | 17 411 (236 individuals) | | |
| **Total number of SNPS and individuals** | | **17 411 (236 individuals)** | | |

**Suppl. Table 1b** Overview of the bio-informatics and SNP filtering for stone loach.

|  |  | **Library 1** | **Library 2** | **Library 3** |
| --- | --- | --- | --- | --- |
| N raw reads | - | 438 482 186 | 444 945 438 | 362 515 442 |
| N reads - Demultiplexing | Barcode Not Found | 1 459 958 (0.3%) | 1 189 364 (0.3%) | 8 493 766 (2.3%) |
|  | Low Quality | 2 169 156 (0.5%) | 820 352 (0.2%) | 481 595 (0.1%) |
|  | RAD Cutsite Not Found | 5 217 272 (1.2%) | 4 051 625 (0.9%) | 19 058 682 (5.3%) |
|  | Retained | 429 635 800 (98.0%) | 438 884 097 (98.6%) | 334 481 399 (92.3%) |
|  | N Individuals | 96 | 95 | 96 |
| N Loci - Stacks | Reference based |  |  |  |
|  | Denovo | 3 572 397 | | |
|  | Populations | 114 819 (287 individuals) | | |
| SNP filtering | Removing duplicated, | 51 073 (287 individuals) | | |
|  | monomorphic and non-common markers |  |  |  |
|  | Removing individuals with missingness | 51 703 (271 individuals) | | |
|  | above 20% |  |  |  |
|  | Heterozygosity markers 0.01-0.5 | 51 703 (271 individuals) | | |
|  | MAC 3 | 35 390 (271 individuals) | | |
|  | Coverage 10-100 | 35 228 (271 individuals) | | |
|  | Marker missing proportion 20% | 35 228 (271 individuals) | | |
|  | SNP read position: based on outliers | 35 228 (271 individuals) | | |
|  | SNP per locus: based on outliers | 34 920 (271 individuals) | | |
|  | Short LD: based on MAC | 17 724 (271 individuals) | | |
|  | Heterozygosity individuals 0.01-0.5 | 17 724 (271 individuals) | | |
|  | Duplicate samples: 0.25 | 17 724 (259 individuals) | | |
|  | HWE | 17 720 (259 individuals) | | |
| **Total number of SNPS and individuals** | | 17 720 (259 individuals) | | |

|  |  | **Library 1** | **Library 2** | **Library 3** |
| --- | --- | --- | --- | --- |
| N raw reads | - | 453 850 732 | 475 667 612 | 494 477 318 |
| N reads - Demultiplexing | Barcode Not Found | 1 310 812 (0.3%) | 2 474 038 (0.5%) | 2 057 704 (0.4%) |
|  | Low Quality | 821 604 (0.2%) | 819 416 (0.2%) | 1 157 035 (0.2%) |
|  | RAD Cutsite Not Found | 4 544 880 (1.0%) | 8 345 326 (1.8%) | 8 223 748 (1.6%) |
|  | Retained | 447 173 436 (98.5%) | 464 028 832 (97.6%) | 483 038 831 (97.7%) |
|  | N Individuals | 96 | 95 | 96 |
| N Loci - Stacks | Reference based |  |  |  |
|  | Denovo | 4 246 501 | | |
|  | Populations | 258 817 (280 individuals) | | |
| SNP filtering | Removing duplicated, | 100 321 (280 individuals) | | |
|  | monomorphic and non-common markers |  |  |  |
|  | Removing individuals with missingness | 100 321 (255 individuals) | | |
|  | above 20% |  |  |  |
|  | Heterozygosity markers 0.01-0.5 | 100 321 (255 individuals) | | |
|  | MAC 3 | 73 431 (255 individuals) | | |
|  | Coverage 10-100 | 72 693 (255 individuals) | | |
|  | Marker missing proportion 20% | 72 693 (255 individuals) | | |
|  | SNP read position: based on outliers | 68 708 (255 individuals) | | |
|  | SNP per locus: based on outliers | 68 132 (255 individuals) | | |
|  | Short LD: based on MAC | 23 407 (255 individuals) | | |
|  | Heterozygosity individuals 0.01-0.5 | 23 407 (255 individuals) | | |
|  | Duplicate samples: 0.25 | 23 407 (249 individuals) | | |
|  | HWE | 23 401 (249 individuals) | | |
| **Total number of SNPS and individuals** | | **23 401 (249 individuals)** | | |

**Suppl. Table 1c** Overview of the bio-informatics and SNP filtering for topmouth gudgeon.


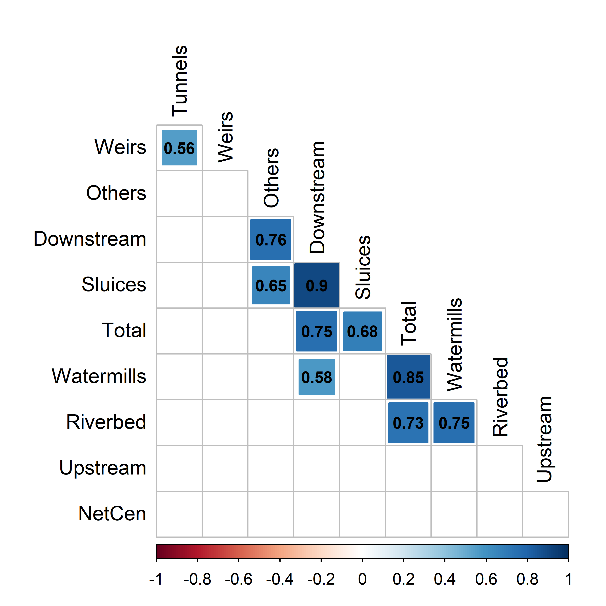


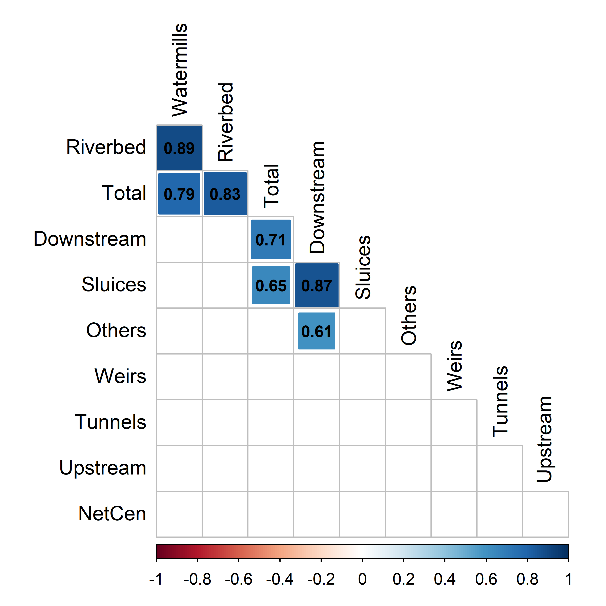


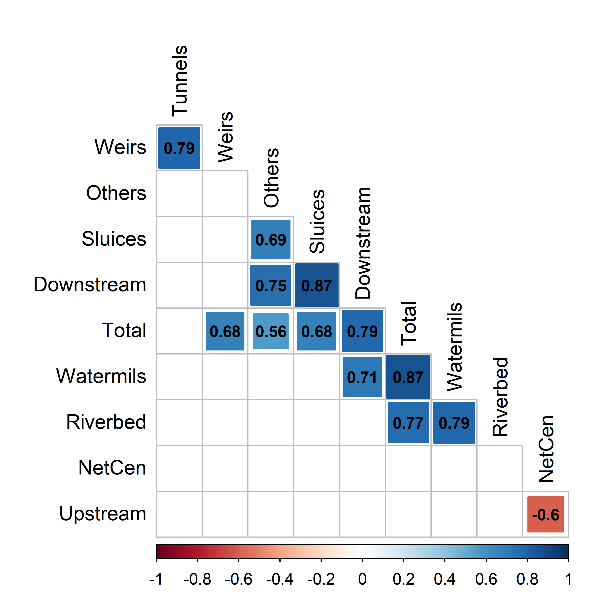


**Suppl. Figure 1** Correlation plot for the spatial variables for three-spined stickleback (top), stone loach (middle), and topmouth gudgeon (bottom). Only significant values (P < 0.05) are shown.

**Suppl. Table 2** Pairwise genetic differentiation based on F_ST_ (Weir & Cockerham, 1984) of three-spined stickleback (below diagonal) and confidence intervals (above diagonal). None of the confidence intervals span zero.

|  | **WingB** | **BegB** | **ZwaP** | **SteT** | **MelR** | **KlhS** | **VelG** | **HerHo** | **KaaD** | **MenT** | **DemT** | **WinR** | **DorL** | **FonT** |
| --- | --- | --- | --- | --- | --- | --- | --- | --- | --- | --- | --- | --- | --- | --- |
| **WingB** |  | 0.1060-0.1353 | 0.1419-0.1785 | 0.0864-0.1142 | 0.0572-0.0904 | 0.0902-0.1156 | 0.0439-0.0753 | 0.1354-0.1668 | 0.0891-0.1193 | 0.08100.1195 | 0.2433-0.2800 | 0.0395-0.0712 | 0.1109-0.1591 | 0.0459-0.1695 |
| **BegB** | 0.1189 |  | 0.1848-0.2161 | 0.0853-0.1141 | 0.0798-0.1121 | 0.0839-0.1125 | 0.0854-0.1181 | 0.1154-0.1522 | 0.0791-0.1048 | 0.1025-0.1416 | 0.2341-0.2635 | 0.0934-0.1230 | 0.1206-0.1778 | 0.0298-0.1825 |
| **ZwaP** | 0.1584 | 0.1972 |  | 0.1580-0.1894 | 0.1283-0.1652 | 0.1612-0.1925 | 0.1346-0.1728 | 0.2073-0.2421 | 0.1598-0.1920 | 0.1666-0.2107 | 0.3134-0.3472 | 0.1255-0.1605 | 0.1901-0.2329 | 0.0667-0.2343 |
| **SteT** | 0.0993 | 0.0979 | 0.1714 |  | 0.0579-0.0901 | 0.0623-0.0863 | 0.0749-0.1060 | 0.0919-0.1282 | 0.0325-0.0546 | 0.0879-0.1293 | 0.1991-0.2271 | 0.0806-0.1072 | 0.1093-0.1562 | 0.0172-0.1421 |
| **MelR** | 0.0718 | 0.0942 | 0.1451 | 0.0747 |  | 0.0612-0.0866 | 0.0482-0.0802 | 0.0947-0.1291 | 0.0582-0.0928 | 0.0676-0.1069 | 0.2013-0.2372 | 0.0462-0.0831 | 0.0960-0.1407 | 0.0072-0.1412 |
| **KlhS** | 0.1021 | 0.0957 | 0.1748 | 0.0732 | 0.0722 |  | 0.0778-0.1169 | 0.0339-0.0704 | 0.0482-0.069 | 0.0727-0.1101 | 0.1821-0.2103 | 0.0832-0.1119 | 0.1018-0.1453 | 0.0266-0.1366 |
| **VelG** | 0.0583 | 0.1015 | 0.1506 | 0.0886 | 0.0623 | 0.0916 |  | 0.1241-0.1631 | 0.0724-0.1094 | 0.0655-0.1110 | 0.2314-0.2710 | 0.0433-0.0779 | 0.1031-0.1506 | 0.0308-0.1698 |
| **HerHo** | 0.1528 | 0.1300 | 0.2237 | 0.1084 | 0.1109 | 0.0509 | 0.1413 |  | 0.0774-0.1107 | 0.1002-0.1403 | 0.2101-0.2405 | 0.1320-0.1649 | 0.1362-0.1830 | 0.0704-0.1803 |
| **KaaD** | 0.1032 | 0.0913 | 0.1741 | 0.0438 | 0.0729 | 0.0581 | 0.0912 | 0.0917 |  | 0.0778-0.1147 | 0.1466-0.1862 | 0.0796-0.1152 | 0.0964-0.1416 | 0.0098-0.1082 |
| **MenT** | 0.0954 | 0.1170 | 0.1828 | 0.1021 | 0.0816 | 0.0855 | 0.0809 | 0.1164 | 0.0927 |  | 0.2177-0.2624 | 0.0772-0.1172 | 0.0898-0.141 | 0.0457-0.1617 |
| **DemT** | 0.2580 | 0.2473 | 0.3290 | 0.2112 | 0.2180 | 0.1956 | 0.2482 | 0.2230 | 0.1648 | 0.2327 |  | 0.2289-0.2661 | 0.2215-0.2652 | 0.1535-0.2418 |
| **WinR** | 0.0552 | 0.1070 | 0.1391 | 0.0916 | 0.0608 | 0.0962 | 0.0580 | 0.1478 | 0.0936 | 0.0932 | 0.2455 |  | 0.1075-0.1510 | 0.0321-0.1569 |
| **DorL** | 0.1320 | 0.1485 | 0.2107 | 0.1329 | 0.1158 | 0.1237 | 0.1242 | 0.1590 | 0.1196 | 0.1137 | 0.2425 | 0.1271 |  | 0.0710-0.1862 |
| **FonT** | 0.0893 | 0.0884 | 0.1410 | 0.0615 | 0.0560 | 0.0647 | 0.0804 | 0.1097 | 0.0476 | 0.0874 | 0.1855 | 0.0758 | 0.1124 |  |

**Suppl. Table 3** Pairwise genetic differentiation based on F_ST_ (Weir and Cockerham, 1984) of stone loach (below diagonal) and confidence intervals (above diagonal). Confidence intervals spanning zero are in bold.

|  | **WingB** | **BegB** | **ZwaP** | **SteT** | **MelR** | **KlhS** | **VelG** | **HerHo** | **KaaD** | **MenT** | **KlbS** | **HerS** | **ZwbB** | **KlegE** |
| --- | --- | --- | --- | --- | --- | --- | --- | --- | --- | --- | --- | --- | --- | --- |
| **WingB** |  | 0.0820-0.1267 | 0.0454-0.0732 | 0.0462-0.0798 | 0.0615-0.1008 | 0.0695-0.0972 | 0.0827-0.1085 | 0.1445-0.1727 | 0.1789-0.2103 | 0.1053-0.1335 | 0.0451-0.0787 | 0.0478-0.0875 | 0.0558-0.0904 | 0.0651-0.0960 |
| **BegB** | 0.1013 |  | 0.0547-0.0871 | 0.0195-0.0494 | 0.0235-0.0538 | 0.0374-0.0672 | 0.1148-0.1454 | 0.1365-0.1701 | 0.1776-0.2113 | 0.0763-0.1095 | 0.0190-0.0516 | 0.0139-0.0544 | 0.0719-0.1181 | 0.0158-0.0458 |
| **ZwaP** | 0.0595 | 0.0708 |  | 0.0266-0.0520 | 0.0417-0.0706 | 0.0407-0.0663 | 0.0822-0.1054 | 0.1171-0.1425 | 0.1523-0.1755 | 0.0820-0.1104 | 0.02020-0.0487 | 0.0249-0.0576 | **-0.008-0.0203** | 0.0382-0.0660 |
| **SteT** | 0.0640 | 0.0323 | 0.0391 |  | **-0.0026-0.0269** | 0.0091-0.0320 | 0.0763-0.1000 | 0.1033-0.1290 | 0.1316-0.1593 | 0.0522-0.0778 | **-0.0097-0.0172** | **-0.0140-0.0195** | 0.0450-0.0735 | **-0.0059-0.0161** |
| **MelR** | 0.0808 | 0.0372 | 0.0537 | 0.0116 |  | 0.0206-0.0454 | 0.0914-0.1163 | 0.1186-0.1452 | 0.1557-0.1851 | 0.0612-0.0909 | 0.0004-0.0269 | **-0.0038-0.0301** | 0.0635-0.0961 | **-0.0009-0.0259** |
| **KlhS** | 0.0827 | 0.0491 | 0.0532 | 0.0188 | 0.0309 |  | 0.0986-0.1194 | 0.0728-0.0960 | 0.1562-0.1794 | 0.0709-0.0925 | 0.0090-0.0314 | 0.0027-0.0357 | 0.0620-0.0926 | 0.0141-0.0343 |
| **VelG** | 0.0946 | 0.1268 | 0.0913 | 0.0849 | 0.1018 | 0.1084 |  | 0.1713-0.1914 | 0.2055-0.2264 | 0.1318-0.1553 | 0.0676-0.0913 | 0.0754-0.0998 | 0.0930-0.1188 | 0.0934-0.1150 |
| **HerHo** | 0.1582 | 0.1506 | 0.1286 | 0.1135 | 0.1304 | 0.0830 | 0.1811 |  | 0.2110-0.2329 | 0.1472-0.1722 | 0.1050-0.1276 | 0.1017-0.1359 | 0.1355-0.1628 | 0.1167-0.1423 |
| **KaaD** | 0.1941 | 0.1910 | 0.1641 | 0.1453 | 0.1708 | 0.1665 | 0.2151 | 0.2219 |  | 0.1745-0.2032 | 0.1401-0.1674 | 0.1365-0.1711 | 0.1665-0.1945 | 0.1572-0.1810 |
| **MenT** | 0.1185 | 0.0911 | 0.0955 | 0.0642 | 0.0748 | 0.0811 | 0.1421 | 0.1581 | 0.1893 |  | 0.0539-0.0795 | 0.0538-0.0822 | 0.1003-0.1286 | 0.0588-0.0823 |
| **KlbS** | 0.0626 | 0.0321 | 0.0325 | 0.0023 | 0.0128 | 0.0190 | 0.0785 | 0.1150 | 0.1533 | 0.0658 |  | **-0.0143-0.0176** | 0.0333-0.0687 | **-0.0044-0.0175** |
| **HerS** | 0.0671 | 0.0316 | 0.0392 | 0.0015 | 0.0121 | 0.0185 | 0.0868 | 0.1145 | 0.1531 | 0.0659 | 0.0011 |  | 0.0428-0.0825 | **-0.0099-0.0220** |
| **ZwbB** | 0.0719 | 0.0917 | 0.0047 | 0.0586 | 0.0754 | 0.0738 | 0.1048 | 0.1474 | 0.1796 | 0.1139 | 0.0506 | 0.0590 |  | 0.0595-0.0871 |
| **KlegE** | 0.0790 | 0.0288 | 0.0504 | 0.0049 | 0.0102 | 0.0232 | 0.1029 | 0.1273 | 0.1696 | 0.0698 | 0.0050 | 0.0043 | 0.0715 |  |

**Suppl. Table 4** Pairwise genetic differentiation based on F_ST_ (Weir and Cockerham, 1984) of topmouth gudgeon (below diagonal) and confidence intervals (above diagonal). Confidence intervals spanning zero are in bold.

|  | **WingB** | **BegB** | **ZwaP** | **SteT** | **MelR** | **KlhS** | **VelG** | **HerHo** | **HerS** | **KlbS** | **DemT** | **WinR** | **RooZ** | **VelK** |
| --- | --- | --- | --- | --- | --- | --- | --- | --- | --- | --- | --- | --- | --- | --- |
| **WingB** |  | 0.0523-0.0857 | 0.0255-0.0548 | 0.0255-0.0574 | 0.0385-0.0781 | 0.0330-0.0643 | 0.0361-0.0689 | 0.1036-0.1332 | 0.0238-0.0587 | 0.0227-0.0547 | 0.0666-0.0988 | **-0.0051-0.414** | 0.0299-0.0610 | 0.0360-0.0668 |
| **BegB** | 0.0668 |  | 0.0278-0.0586 | 0.0208-0.0541 | 0.0439-0.0779 | 0.0353-0.0647 | 0.0411-0.0690 | 0.1017-0.1386 | 0.0278-0.0613 | 0.0139-0.0564 | 0.0714-0.1044 | 0.0346-0.0841 | 0.0265-0.0562 | 0.0379-0.0658 |
| **ZwaP** | 0.0389 | 0.0411 |  | **-0.0048-0.0239** | 0.0133-0.0519 | 0.0038-0.0263 | 0.0058-0.303 | 0.0705-0.1029 | **-0.0008-0.0239** | **-0.0057-0.0250** | 0.0474-0.0774 | 0.004-0.0485 | 0.0006-0.0275 | 0.0043-0.0309 |
| **SteT** | 0.0404 | 0.0357 | 0.0080 |  | 0.0053-0.485 | **-0.0055-0.0219** | 0.0017-0.0286 | 0.0701-0.1074 | **-0.0120-0.0175** | **-0.103-0.0150** | 0.0423-0.0752 | 0.0038-0.0437 | **-0.0064-0.0211** | 0.0012-0.0279 |
| **MelR** | 0.0546 | 0.0578 | 0.0302 | 0.0232 |  | 0.0165-0.0501 | 0.0226-0.0554 | 0.0881-0.1222 | **-0.0100-0.0440** | **-0.0120-0.0458** | 0.0571-0.0924 | 0.0202-0.0683 | 0.149-0.0460 | 0.0224-0.0552 |
| **KlhS** | 0.0476 | 0.0474 | 0.0137 | 0.0076 | 0.0317 |  | 0.0015-0.0309 | 0.0414-0.0841 | **-0.0061-0.0216** | **-0.0024-0.0228** | 0.0480-0.0752 | 0.0131-0.0517 | 0.0020-0.0282 | 0.0005-0.0296 |
| **VelG** | 0.0500 | 0.0521 | 0.0167 | 0.0137 | 0.0369 | 0.0134 |  | 0.0800-0.1127 | 0.0014-0.0280 | 0.0009-0.0306 | 0.0563-0.0856 | 0.0177-0.0581 | 0.0010-0.0289 | **-0.0073-0.0194** |
| **HerHo** | 0.1162 | 0.1182 | 0.0840 | 0.0845 | 0.1035 | 0.0608 | 0.0946 |  | 0.0651-0.0987 | 0.0697-0.1023 | 0.1230-0.1561 | 0.0818-0.1268 | 0.0712-0.1055 | 0.0975-0.1075 |
| **HerS** | 0.0402 | 0.0404 | 0.0103 | 0.0004 | 0.0259 | 0.0055 | 0.0135 | 0.0788 |  | **-0.0072-0.0190** | 0.0415-0.0726 | 0.0048-0.0475 | **-0.0042-0.0239** | 0.0005-0.0285 |
| **KlbS** | 0.0365 | 0.0319 | 0.0063 | 0.0017 | 0.0248 | 0.0089 | 0.0130 | 0.0826 | 0.0045 |  | 0.0414-0.0714 | 0.0018-0.0422 | **-0.0056-0.0223** | 0.0014-0.0285 |
| **DemT** | 0.0823 | 0.0860 | 0.0610 | 0.0577 | 0.0722 | 0.0601 | 0.0706 | 0.1372 | 0.0549 | 0.0537 |  | 0.0522-0.0964 | 0.0501-0.0800 | 0.0555-0.0837 |
| **WinR** | 0.0118 | 0.0510 | 0.0215 | 0.0214 | 0.0395 | 0.0284 | 0.0324 | 0.0993 | 0.0225 | 0.0193 | 0.0692 |  | 0.0055-0.0502 | 0.0162-0.0553 |
| **RooZ** | 0.0421 | 0.0389 | 0.0110 | 0.0059 | 0.0300 | 0.0128 | 0.0137 | 0.0852 | 0.0073 | 0.0065 | 0.0638 | 0.0226 |  | 0.0010-0.0290 |
| **VelK** | 0.0501 | 0.0492 | 0.0163 | 0.0132 | 0.0377 | 0.0125 | 0.0050 | 0.0920 | 0.0114 | 0.0124 | 0.0694 | 0.0319 | 0.0137 |  |

|  |  | **three-spined stickleback** | | | **stone loach** | | **topmouth gudgeon** | |
| --- | --- | --- | --- | --- | --- | --- | --- | --- |
|  |  | Simple | Partial | Simple | | Partial | Simple | Partial |
| Riverbed obstructions | r | 0.0424 | -0.0117 | 0.4391 | | 0.3261 | **0.6490** | **0.6072** |
|  | P | 0.3730 | 0.4640 | 0.1070 | | 0.1550 | **0.0230** | **0.0440** |
| Weirs | r | 0.2336 | 0.1382 | -0.0084 | | -0.0042 | 0.3259 | 0.4322 |
|  | P | 0.1570 | 0.1650 | 0.4230 | | 0.4210 | 0.1730 | 0.1510 |
| Watermills | r | 0.1398 | 0.0646 | **0.6064** | | 0.4398 | **0.6346** | **0.4589** |
|  | P | 0.2430 | 0.3340 | **0.0360** | | 0.0850 | **0.0320** | **0.1280** |
| Tunnels | r | -0.0164 | -0.0348 | 0.0224 | | -0.0487 | 0.4729 | 0.5293 |
|  | P | 0.4590 | 0.4790 | 0.4150 | | 0.4580 | 0.0830 | 0.0780 |
| Others | r | 0.3184 | 0.2772 | -0.0085 | | -0.4894 | 0.2384 | -0.1929 |
|  | P | 0.1700 | 0.2170 | 0.4780 | | 0.9210 | 0.2550 | 0.6910 |
| Sluices | r | 0.0006 | -0.2157 | 0.0882 | | -0.7362 | 0.3538 | -0.3318 |
|  | P | 0.4300 | 0.7910 | 0.3010 | | 1.0000 | 0.1240 | 0.8330 |
| Total | r | 0.1439 | 0.0649 | 0.4035 | | 0.1272 | **0.7493** | **0.6182** |
|  | P | 0.3360 | 0.4160 | 0.0740 | | 0.3270 | **0.0010** | **0.0180** |

**Suppl. Table 5** Results of the simple and partial mantel tests correlating genetic distance and distance based on pairwise number of barriers only including the eight overlapping sampling locations. Significant values are in bold.


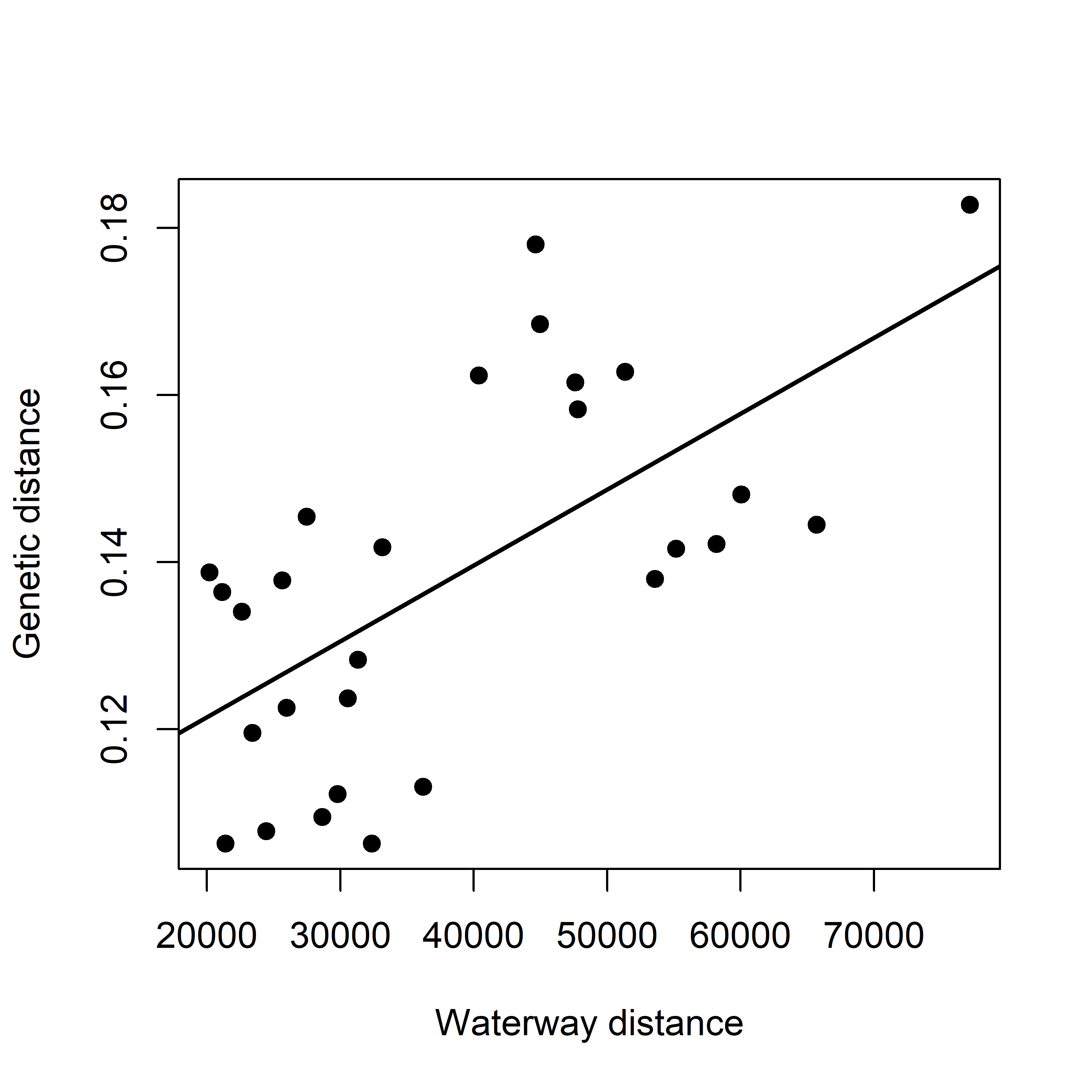

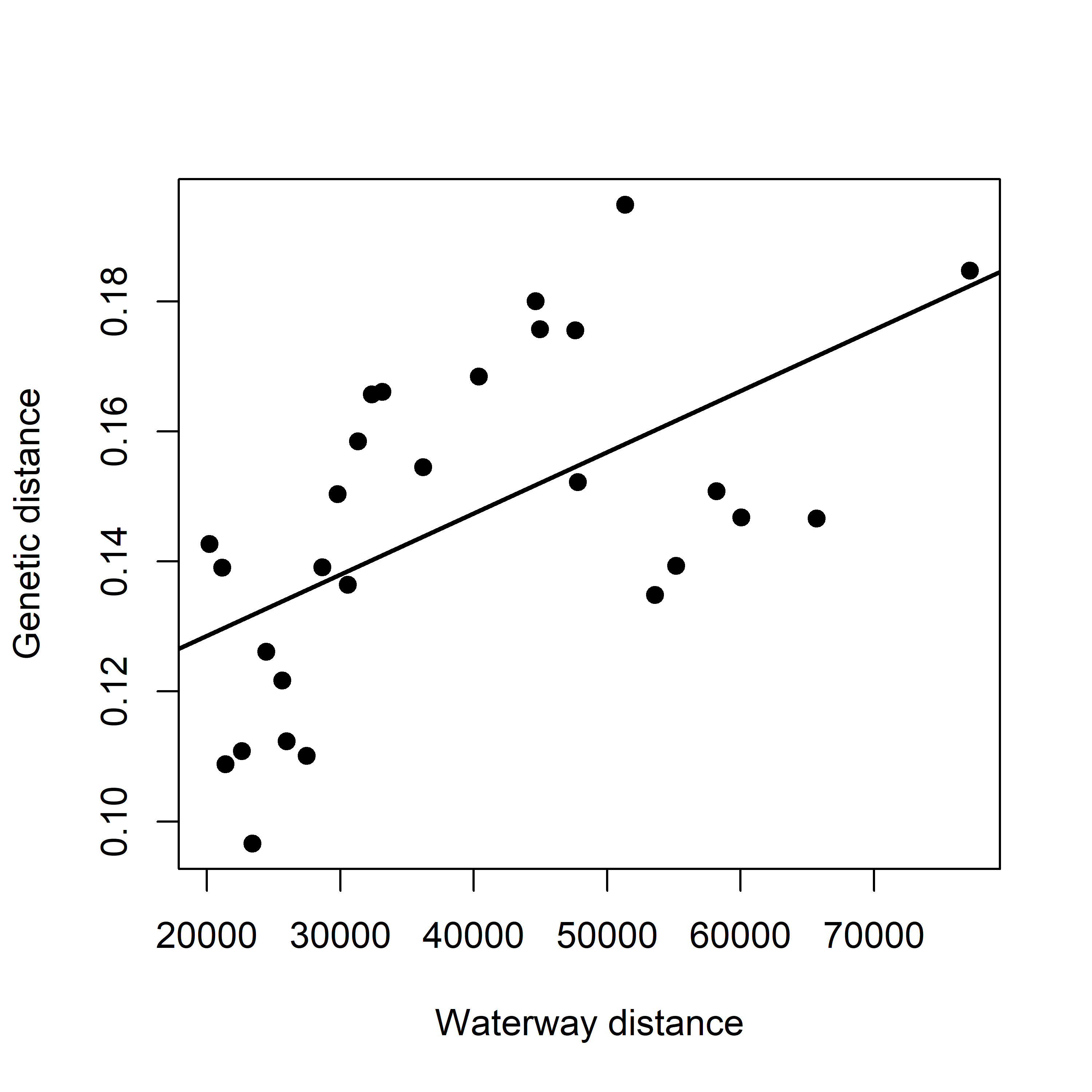


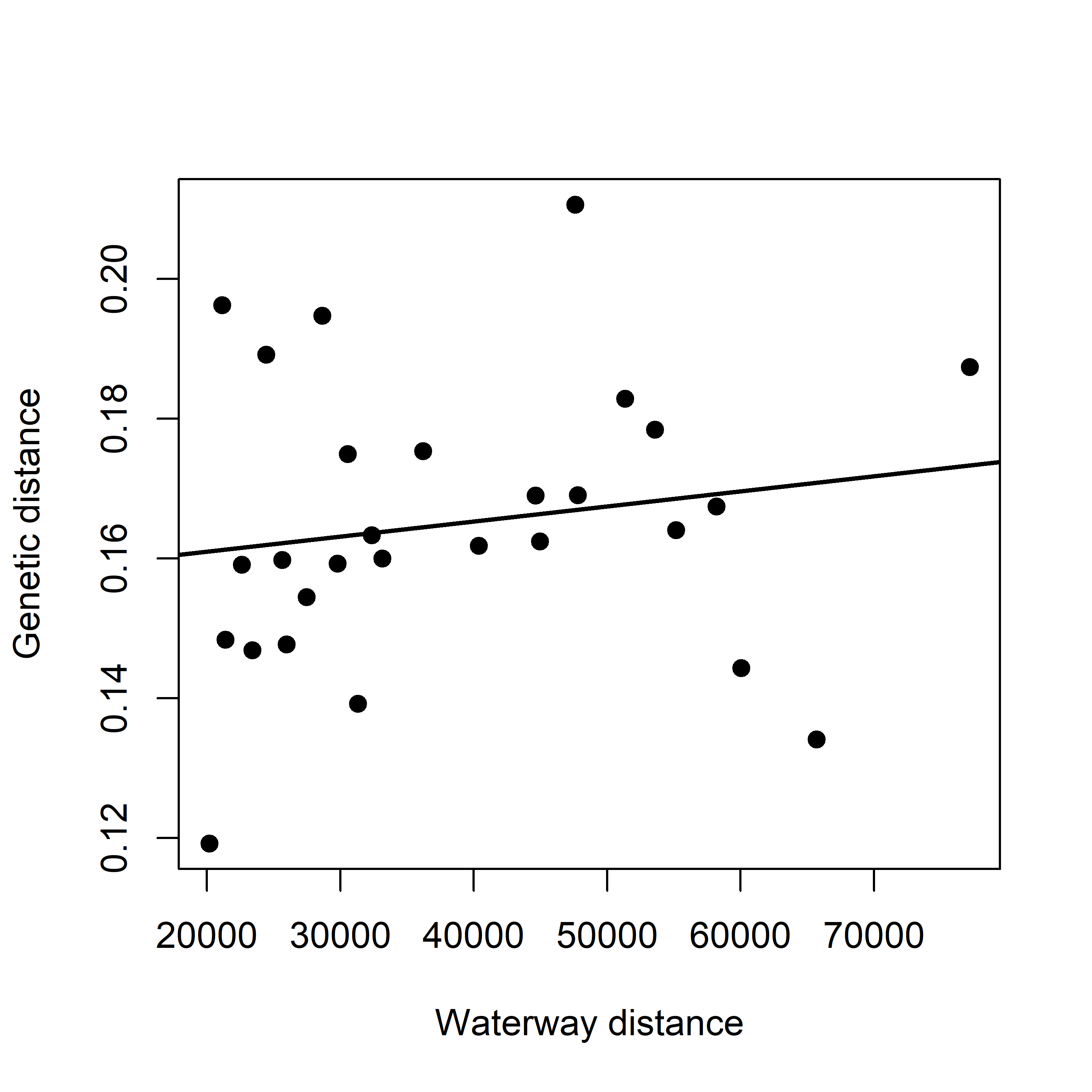


**Suppl. Figure 2** Isolation-By-Distance plot based on pairwise waterway distances for the eight overlapping sampling location for three-spined stickleback (left), stone loach (middle), and topmouth gudgeon (right).

**Suppl. Table 6** Results for the redundancy analysis for the eight overlapping sampling sites.

|  |  | **three-spined stickleback** | | | **stone loach** | | | **yopmouth gudgeon** | | |
| --- | --- | --- | --- | --- | --- | --- | --- | --- | --- | --- |
|  |  | P | F | Variance | P | F | Variance | P | F | Variance |
| Physico-chemistry | Temperature | 0.001 | 6.556 | 0.001 | 0.001 | 4.915 | 0.001 | 0.001 | 2.467 | 0.001 |
|  | Oxygen | 0.001 | 5.585 | 0.002 | 0.001 | 5.895 | 0.001 | 0.001 | 2.342 | 0.001 |
|  | Conductivity | 0.001 | 6.696 | 0.001 | 0.001 | 7.477 | 0.002 | 0.001 | 3.405 | 0.001 |
|  | Nitrogen | 0.001 | 7.561 | 0.002 | 0.001 | 6.000 | 0.001 | 0.001 | 3.547 | 0.001 |
|  | Phosphorus | 0.001 | 5.348 | 0.001 | 0.001 | 8.479 | 0.002 | 0.001 | 2.435 | 0.001 |
|  | Density | 0.001 | 6.655 | 0.001 | 0.001 | 3.582 | 0.008 | 0.001 | 1.978 | 0.001 |
| Space | PCNM1 | - | - | - | 0.001 | 7.673 | 0.002 | 0.001 | - | - |
|  | PCNM2 | 0.001 | 6.824 | 0.002 | - | - | - | - | 2.560 | 0.007 |
|  | PCNM3 | - | - | - | 0.001 | 4.017 | 0.001 | 0.001 | 2.175 | 0.001 |
|  | Network centrality | 0.001 | 7.384 | 0.002 | 0.001 | 3.635 | 0.001 | 0.001 | 3.288 | 0.001 |
|  | Upstream distance | 0.001 | 5.337 | 0.116 | 0.001 | 2.063 | 0.001 | 0.001 | 2.544 | 0.001 |
|  | Downstream distance | 0.001 | 7.420 | 0.002 | 0.001 | 5.536 | 0.001 | 0.001 | 3.909 | 0.001 |
| Barriers | Tunnels | 0.001 | 5.328 | 0.001 | 0.001 | 5.648 | 0.001 | 0.001 | 3.969 | 0.001 |
|  | Sluices | 0.001 | 5.051 | 0.001 | 0.001 | 3.984 | 0.001 | 0.001 | 3.984 | 0.001 |
|  | Weirs | 0.001 | 4.280 | 0.001 | 0.001 | 7.331 | 0.002 | - | - | - |
|  | Watermills | 0.001 | 10.964 | 0.002 | 0.001 | 9.883 | 0.002 | 0.001 | 4.313 | 0.001 |
|  | RivObs | 0.001 | 9.973 | 0.002 | - | - | - | 0.001 | 5.016 | 0.001 |


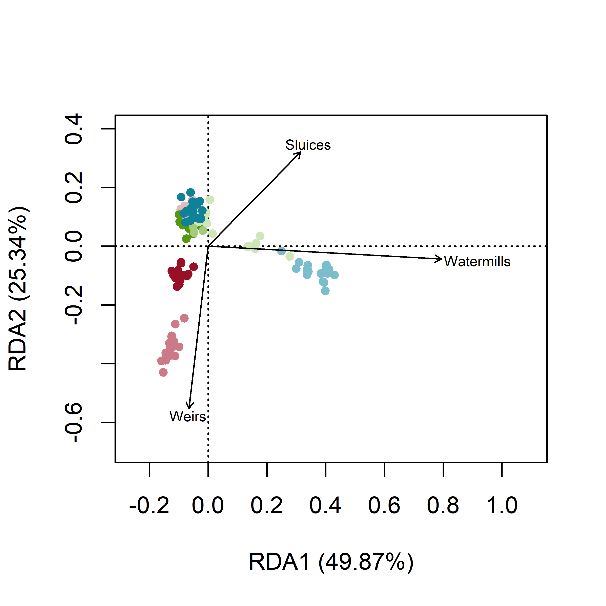

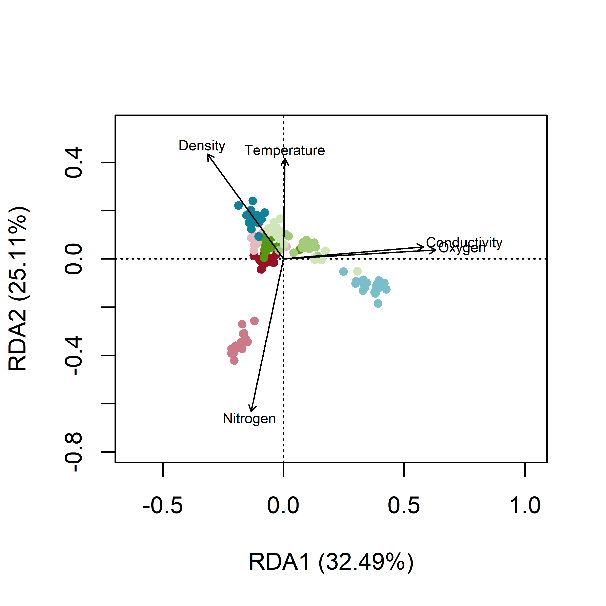

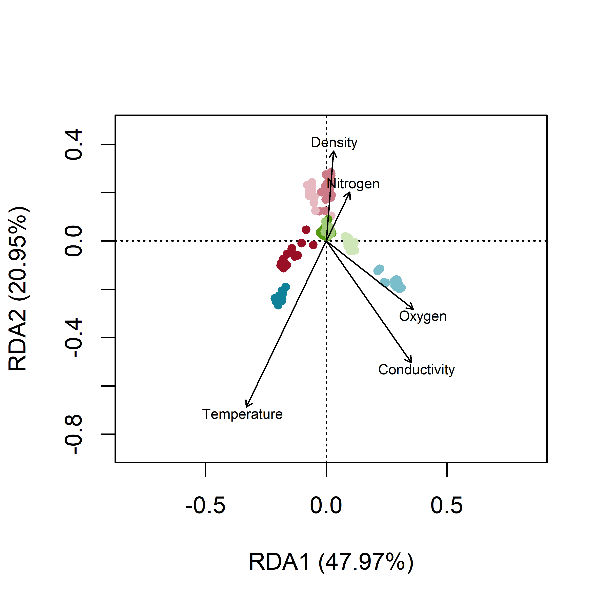

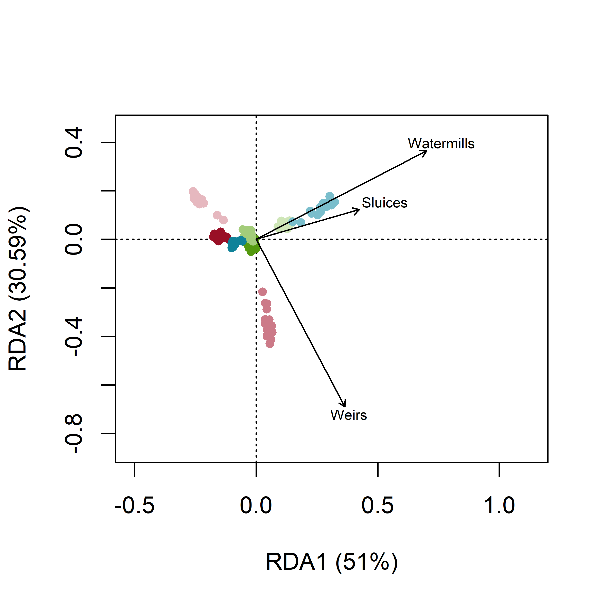

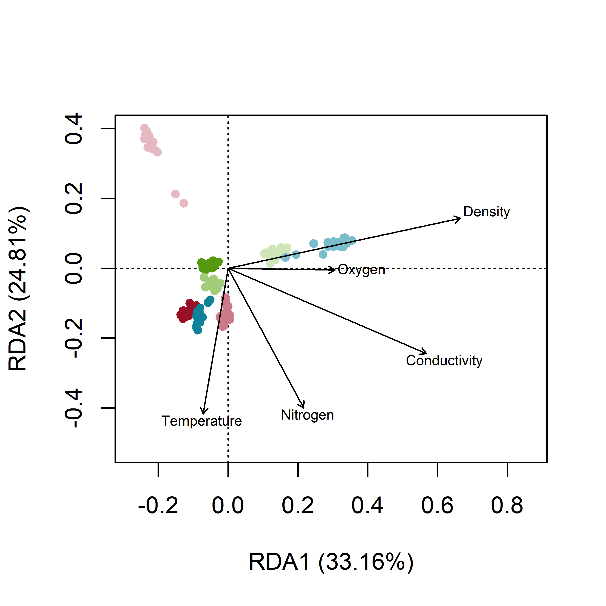


(a)


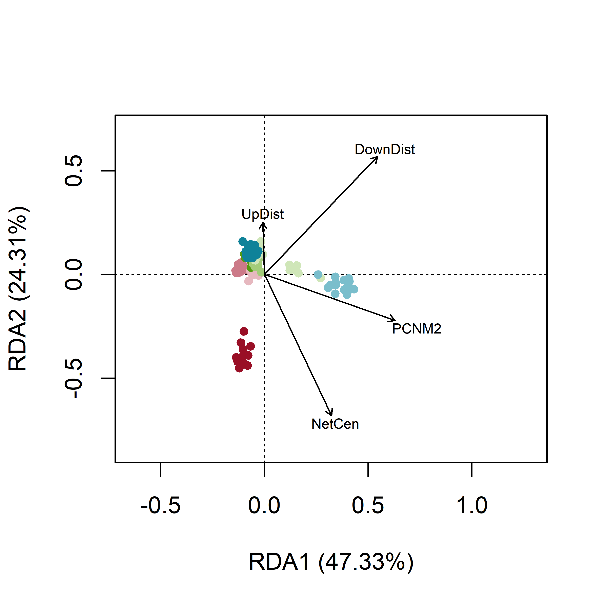

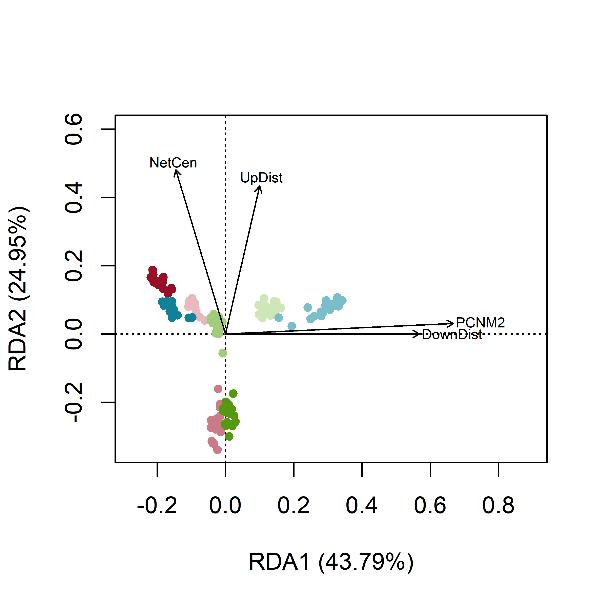


(b)

(c)


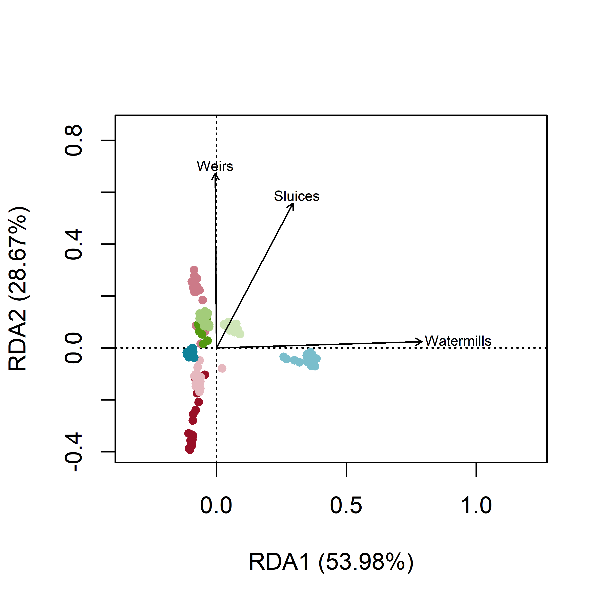

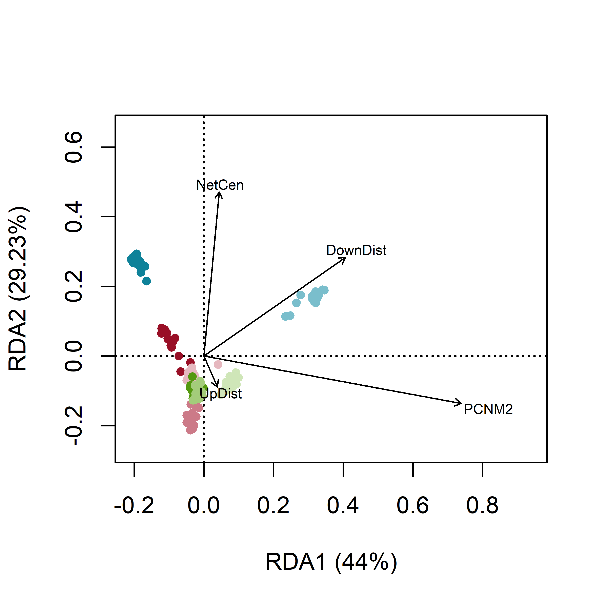


(d)

(e)

(f)

(g)

(h)

(i)

**Figure S3.** Redundancy analysis (RDA) biplot linking the physico-chemical, spatial, and barrier dataset to genetic composition of three-spined stickleback (A-C), stone loach (D-F), and topmouth gudgeon (G-I) for the eight overlapping locpond to different population (see Fig. 2 for the legend).

**Suppl. Figure 3.** Redundancy analysis (RDA) biplot linking the physico-chemical, spatial, and barrier dataset to genetic composition of three-spined stickleback (a-c), stone loach (d-f), and topmouth gudgeon (f-i) for the eight overlapping locations. Colours respond to different population (see Fig. 5.2 for the legend).
